# Quantum Measurements with, and Yet without an Observer

**DOI:** 10.3390/e22101185

**Published:** 2020-10-21

**Authors:** Dmitri Sokolovski

**Affiliations:** 1 Departmento de Química-Física, Universidad del País Vasco, UPV/EHU, 48940 Leioa, Bizkaia, Spain; dgsokol15@gmail.com; 2 IKERBASQUE, Basque Foundation for Science, Plaza Euskadi 5, 48009 Bilbao, Bizkaia, Spain

**Keywords:** quantum mechanics, quantum measurements, quantum interference, Feynman’s paths, Wigner’s friend problem

## Abstract

It is argued that Feynman’s rules for evaluating probabilities, combined with von Neumann’s principle of psycho-physical parallelism, help avoid inconsistencies, often associated with quantum theory. The former allows one to assign probabilities to entire sequences of hypothetical Observers’ experiences, without mentioning the problem of wave function collapse. The latter limits the Observer’s (e.g., Wigner’s friend’s) participation in a measurement to the changes produced in material objects, thus leaving his/her consciousness outside the picture.

## 1. Introduction

Recently there was a renewed interest in whether quantum theory is internally consistent in its present form, or if new assumptions need to be added to its already well established principles. The discussion initiated by the authors of [1] was quickly joined, and various opinions were expressed [2,3,4,5,6,7,8,9,10,11]. An analysis often centres on two issues, the “collapse” of the quantum state, and the role and place of a conscious Observer. The two problems are related. The wave function of the observed system is supposed to undergo a sudden change once a definite result of the observation becomes known to the Observer. This change, reminiscent of what happens to a probability distribution in classical statistics once additional information is received, may have something to do with Observer’s consciousness. A related question is how an Observer, taking part in the experiment, should consider other intelligent participants, and whether his/her reasoning would depend on availability of the information about other Observers’ outcomes [1], or merely on being aware of the other measurements being made. One extreme view includes consciousness into a quantum mechanical calculation directly [12], or grants it an active role in the reshaping of the collapsed wave function [13]. On the other extreme, one finds theories aiming at denying the Observer any special status at all as happens, for example, in the consistent histories approach (CHA) [5,14]. One cannot help wishing for a compromise position. Would it be possible to have a universal quantum theory centred on the Observer’s subjective perceptions, and yet applying its mathematical apparatus only to material objects, whenever Observer’s probability are calculated? One might look for an answer in the literature.

The question was discussed by Bohr [15] and later by von Neumann in his monograph [16], both invoking the principle of psycho-physical parallelism. The principle establishes a correspondence between “extra-physical process of subjective perception” and “equivalent physical processes”, as described by the Observer’s theory. This is a delicate balancing act. According to von Neumann [16], its success depends not on providing a detailed explanation of the act of human perception, but on being able to move the boundary between “physical” and “extra-physical” in an arbitrary manner, deeper into the Observer’s body, or further out towards the observed system. Von Neumann’s discussion covered mostly a single measurement, made on a system in an already known quantum state.

However, current discussions (see, e.g., [1,2,3,4,5,6,7,8,9,10,11]) often consider several consecutive observations, which involve more than one Observer. An approach to such situations was outlined in Feynman’s undergraduate text [17], rarely mentioned in the present context. Feynman’s *general principles* [17] are quite simple. To find the probability of a sequence of observed events, one needs to evaluate the amplitude for each route, by multiplying the amplitudes for each part of the route, add up the amplitudes, if the routes cannot be told apart, and take the absolute square of the sum. Feynman warns against thinking *“in terms of ‘particle waves’”*, and his recipe does not need to address the “collapse”’ problem. Nor is the role of consciousness discussed in great detail. Section 2.6 of [17] hints at the importance of the “traces left” by a phenomenon, and leaves the problem in that form.

The purpose of this paper is to establish whether the principles of [16,17] are sufficient to make an intelligent Observer a client and a beneficiary of quantum theory, at the same time keeping the subject of consciousness outside the theory’s scope. We will also ask whether, with the first task achieved, the theory is able to provide unequivocal answers in the situations where its consistency is questioned.

In Section 2.1 we adopt Feynman’s recipe [17] to describe a series of consecutive quantum measurements. In Section 2.2 we demonstrate the equivalence between this “static” view, and a “evolutionist” picture, in which an initial quantum state is seen as undergoing a unitary evolution, interrupted by Observers’ interventions. Section 2.3 underlines a distinction between Observer’s consciousness, and his/her material memory, thus setting a framework for our analysis. In Section 2.4 we consider the case of two Observers, and three possible scenarios for their experiment. In Section 2.5 we summarise our preliminary conclusions. Section 2.6 revisits the Wigner’s friend problem of Reference [13]. In Section 2.7 we discuss an interference experiment, similar to that proposed in [18]. In Section 2.8 we show how the von Neumann boundary can be placed “at the level of the observed system” in a general case. Section 2.9 describes a more efficient way to calculate the probabilities, in which all but the last Observers are represented by their unobserved probes. In Section 2.10 we discuss certain similarities and differences between our analysis, and the consistent histories approach of [14]. Section 3 contains our conclusions.

## 2. Results

### 2.1. Quantum Rules—The “Entire History” View

Let us assume that L+1 Observers decide to make L+1 measurements on particular parts of a quantum system, with which they associate an *N*-dimensional Hilbert space. If L+1 quantities $Q^{\ell }$, ℓ=0,1,2,…,L, need to be measured at different times $t=t_{\ell }$, $t_{\ell }>t_{\ell -1}$, one associates with each $Q^{\ell }$ a discrete orthonormal basis $|q_{n_{\ell }}^{\ell }\rangle$, $n_{\ell }=1,\ldots N$, and a Hermitian operator, ${\hat{Q}}^{\ell }$, of which the eigenvalues $Q_{m_{\ell }}^{\ell }$, $m_{\ell }=1\ldots M_{\ell }$ may be degenerate, $M_{\ell }\leq N$,

(1) $$\begin{array}{c} {\hat{Q}}^{\ell }=\sum_{m_{\ell }=1}^{M_{\ell }}Q_{m_{\ell }}^{\ell }{\hat{\Pi }}_{m_{\ell }}^{\ell },\;{\hat{\Pi }}_{m_{\ell }}^{\ell }\equiv \sum_{n_{\ell }=1}^{N}\Delta \left(Q_{m_{\ell }}^{\ell }-\langle q_{n_{\ell }}^{\ell }\left|{\hat{Q}}^{\ell }\right|q_{n_{\ell }}^{\ell }\rangle \right)\left|q_{n_{\ell }}^{\ell }\rangle \langle q_{n_{\ell }}^{\ell }\right|. \end{array}$$

We define Δ(x−y)≡1 for x=y, and 0 otherwise, so that a ${\hat{\Pi }}_{m_{\ell }}^{\ell }$ projects onto the eigen-subspace of the eigenvalue $Q_{m_{\ell }}^{\ell }$. Observers’ outcomes must coincide with the eigenvalues of the operators ${\hat{Q}}^{\ell }$, and one wishes to evaluate the probabilities $P(Q_{m_{L}}^{L}\ldots \leftarrow Q_{m_{\ell }}^{\ell }\ldots \leftarrow Q_{m_{0}}^{0})$ of obtaining a series of outcomes $Q_{m_{L}}^{L}\ldots \leftarrow Q_{m_{\ell }}^{\ell }\ldots \leftarrow Q_{m_{0}}^{0}$. The initial measurement (also known as the preparation) must determine the initial state $|q_{i_{1}}^{1}\rangle$ unambiguously, $Q_{m_{0}}^{0}↔|q_{n_{0}}^{0}\rangle$, and we will always assume that $Q_{m_{0}}^{0}$ is non-degenerate.

The recipe for constructing the probabilities $P(Q_{m_{L}}^{L}\ldots \leftarrow Q_{m_{\ell }}^{\ell }\ldots .\leftarrow Q_{m_{0}}^{0})$ is as follows [17]. First, one constructs all *virtual* (Feynman) paths, $\left\{q_{n_{L}}^{L}\ldots \leftarrow q_{n_{\ell }}^{\ell }\ldots \leftarrow q_{n_{0}}^{0}\right\}$, connecting the eigenstates $|q_{i_{\ell }}^{\ell }\rangle$, and ascribes to them probability amplitudes

(2) $$\begin{array}{c} A\left(q_{n_{L}}^{L}\ldots \leftarrow q_{n_{\ell }}^{\ell }\leftarrow q_{n_{0}}^{0}\right)=\prod_{\ell =1}^{L}\langle q_{n_{\ell }}^{\ell }\left|\hat{U}\left(t_{\ell },t_{\ell -1}\right)\right|q_{n_{\ell -1}}^{\ell -1}\rangle . \end{array}$$

where $\hat{U}\left(t^{\prime },t\right)$ is the system’s evolution operator.

Then one sums the amplitudes in Equation (2) over the degeneracies of all but the last eigenvalues, thus obtaining the “real” paths, $\left\{q_{n_{L}}^{L}\ldots \leftarrow Q_{m_{\ell }}^{\ell }\ldots \leftarrow q_{n_{0}}^{0}\right\}$, endowed with the *probability amplitudes*

(3) $$\begin{array}{c} \tilde{A}\left(q_{n_{L}}^{L}\ldots \leftarrow Q_{m_{\ell }}^{\ell }\ldots \leftarrow q_{n_{0}}^{0}\right)=\sum_{n_{1},,\ldots n_{L-1}=1}^{N}\left[\prod_{\ell =1}^{L-1}\Delta \left(Q_{m_{\ell }}^{\ell }-\langle q_{n_{\ell }}^{\ell }\left|{\hat{Q}}^{\ell }\ell \right|q_{n_{\ell }}^{\ell }\rangle \right)\right]A\left(q_{n_{L}}^{L}\ldots \leftarrow q_{n_{\ell }}^{\ell }\ldots \leftarrow q_{i_{0}}^{0}\right),\; \end{array}$$

as well as the *probabilities*

(4) $$\begin{array}{c} p\left(q_{n_{L}}^{L}\ldots \leftarrow Q_{m_{\ell }}^{\ell }\ldots \leftarrow q_{n_{0}}^{0}\right)\equiv {\left|\tilde{A}\left(q_{n_{L}}^{L}\ldots \leftarrow Q_{m_{\ell }}^{\ell }\ldots \leftarrow q_{n_{0}}^{0}\right)\right|}^{2}. \end{array}$$

Finally, one sums the probabilities in Equation (4) over the degeneracies of the last ${\hat{Q}}^{L}$, to obtain

(5) $$\begin{array}{c} P\left(Q_{m_{L}}^{L}\ldots \leftarrow Q_{m_{\ell }}^{\ell }\ldots .\leftarrow Q_{m_{0}}^{0}\right)=\sum_{n_{L}=1}^{N}\Delta \left(Q_{m_{L}}^{L}-\langle q_{n_{L}}^{L}\left|{\hat{Q}}^{L}\right|q_{n_{L}}^{L}\rangle \right)p\left(q_{n_{L}}^{L}\ldots \leftarrow Q_{m_{\ell }}^{\ell }\ldots \leftarrow q_{n_{0}}^{0}\right) \end{array}$$

In general, the situation is non-Markovian—the probability $p(q_{n_{L}}^{L}\ldots \leftarrow Q_{m_{\ell }}^{\ell }\ldots \leftarrow q_{n_{0}}^{0})$ does not factorise into $\prod_{\ell =1}^{L}p\left(Q_{m_{\ell }}^{\ell }-Q_{m_{\ell -1}}^{\ell -1}\right)$, unless all the eigenvalues are non-degenerate, $M_{\ell }=N$. For this reason, the amplitude $\tilde{A}\left(q_{n_{L}}^{L}\ldots \leftarrow Q_{m_{\ell }}^{\ell }\ldots \leftarrow q_{n_{0}}^{0}\right)$ has to refer to the *entire* experiment, which starts with the preparation at $t=t_{0}$, and ends with the last observation made at $t=t_{L}$.

Finally, we need to assume that the probabilities (5) refer to the Observers’ experiences, and not to the statements like “a physical quantity has a certain value” [16]. The situation should, therefore, be like this. In an experiment, involving several steps, each participant can perceive one of his/her possible outcomes, $Q_{m_{\ell }}^{\ell }$. Equations (2)–(5) give a recipe for calculating the likelihoods of all possible sequences of the perceived outcomes *one at a time*. The recipe consists in calculating matrix elements of unitary operators, multiplying the results, and adding the products, as appropriate. There is no mention of a “state evolving throughout experiment”, neither a need to account for the future development of such a state, after the experiment is finished at $t=t_{L}$. One does not need to care about what the participants may think or know about each other. The calculation could be made by an Alice, who does not take part in the experiment, and remains in the comfort of her office. Her results will apply to any L+1 Observers who may or may not communicate with each other, as well as to the same Observer, who performs all L+1 measurements single handedly. However, the problem can also be formulated in a different manner.

### 2.2. Quantum Rules—The “Evolutionist” View

Equation (5) can be written in a more familiar way. Defining a partial evolution operator as

(6) $$\begin{array}{c} \hat{U}\left(Q_{m_{L-1}}^{L-1}\ldots \leftarrow Q_{m_{\ell }}^{\ell }\ldots .\leftarrow q_{m_{0}}^{0}\right)\equiv \hat{U}\left(t_{L},t_{L-1}\right)\prod_{\ell =1}^{L-1}{\hat{\Pi }}_{m_{\ell }}^{\ell }\hat{U}\left(t_{\ell },t_{\ell -1}\right), \end{array}$$

with a property that

(7) $$\begin{array}{c} \sum_{m_{L-1}\ldots m_{1}=1}^{M_{L-1}\ldots M_{1}}\hat{U}\left(Q_{m_{L-1}}^{L-1}\ldots \leftarrow Q_{m_{\ell }}^{\ell }\ldots .\leftarrow Q_{m_{1}}^{1}\right)=\hat{U}\left(t_{L},t_{0}\right),\;\;\;\;\;\; \end{array}$$

and a projector onto the initial state, ${\hat{\rho }}_{0}\equiv \left|q_{n_{0}}^{0}\rangle \langle q_{n_{0}}^{0}\right|$, one can construct a family of $M_{1}\times M_{2}\;\ldots \times M_{L-1}$ density operators (mixed states)

(8) $$\begin{array}{c} \rho (Q_{m_{L-1}}^{L-1}\ldots \leftarrow Q_{m_{\ell }}^{\ell }\ldots .\leftarrow Q_{m_{1}}^{1},\rho_{0})\equiv \;\;\;\;\;\;\\ \hat{U}\left(Q_{m_{L-1}}^{L-1}\ldots \leftarrow Q_{m_{\ell }}^{\ell }\ldots .\leftarrow Q_{m_{1}}^{1}\right){\hat{\rho }}_{0}{\hat{U}}^{†}\left(Q_{m_{L-1}}^{L-1}\ldots \leftarrow Q_{m_{\ell }}^{\ell }\ldots .\leftarrow Q_{m_{1}}^{1}\right), \end{array}$$

where ${\hat{U}}^{†}$ is the hermitian conjugate of $\hat{U}$. Now the probabilities in Equation (5) can be obtained just as well by taking a trace,

(9) $$\begin{array}{c} P\left(Q_{m_{L}}^{L}\ldots \leftarrow Q_{m_{\ell }}^{\ell }\ldots .\leftarrow Q_{m_{0}}^{0}\right)=\mathrm{tr}\left[{\hat{\Pi }}_{m_{L}}^{L}\rho \left(Q_{m_{L-1}}^{L-1}\ldots \leftarrow Q_{m_{\ell }}^{\ell }\ldots .\leftarrow Q_{m_{1}}^{1},\rho_{0}\right)\right] \end{array}$$

Equation (9) remains valid for a more general initial state,

(10) $$\begin{array}{c} \rho_{0}=\sum_{\nu }w_{\nu }\left|q_{0}^{\nu }\rangle \langle q_{0}^{\nu }\right|, \end{array}$$

where the system is prepared in a state $|q_{0}^{\nu }\rangle$ with a probability $w_{\nu }$, and $|q_{0}^{\nu }\rangle$ are any normalised, yet not necessarily orthogonal states.

This is a dynamic picture. In Equation (8) the initial state (9) can be seen as evolving until the time of the last observation, yet the evolution is not unitary,

(11) $$\begin{array}{c} {\hat{U}}^{†}\left(Q_{m_{L-1}}^{L-1}\ldots \leftarrow Q_{m_{\ell }}^{\ell }\ldots .\leftarrow Q_{m_{1}}^{1}\right)\hat{U}\left(Q_{m_{L-1}}^{L-1}\ldots \leftarrow Q_{m_{\ell }}^{\ell }\ldots .\leftarrow Q_{m_{1}}^{1}\right)=\\ {\hat{\Pi }}_{m_{1}}^{1†}\left(t_{1}\right)\ldots \times {\hat{\Pi }}_{m_{L-1}}^{(L-1)†}{\hat{\Pi }}_{m_{L-1}}^{L-1}\ldots \times {\hat{\Pi }}_{m_{1}}^{1}\left(t_{1}\right)\neq \hat{1}.\;\;\;\;\;\; \end{array}$$

In total, there are $M_{1}\times M_{2}\ldots \times M_{L-1}$ such evolutions, and for someone who wishes to associate quantum mechanics with uninterrupted unitary evolution in Equation (7), Equations (9)–(11) may present a conceptual problem. A wave function seen as a substance in continuous flow (7), decimated each time a conscious Observer makes an enquiry and perceives an outcome, presents a rather bizarre picture.

This problem does not arise in the “static” view, outlined at the end of the previous section. In the following, we will accept the rules of Section 2.1 as the basic axioms of quantum theory, and treat Equations (9)–(11) as their consequences [19], which can be derived and used, for example, for computational convenience.

### 2.3. Consciousness, Memory and Material Records

In the context of the previous Section, it is only natural to wonder how an act of perceiving an outcome could succeed in replacing a unitary evolution (7) by a non-unitary one in Equation (11). With Observer’s consciousness now drawn into the discussion, there are at least two possibilities. One can

(i) include Observer’s consciousness into the scope of quantum theory [12]. This is known to lead to a contradiction with what we seem to know about intelligent beings [13].
(ii) Exclude consciousness from the analysis completely, reduce its role to that of an external client, and treat the content of Section 2.1 as a rule book, with no obligation to give any explanations. This appears to be in line with the approach outlined in [17], and articulated in more detail in [19].

A further insight can, however, be gained at the cost of making additional assumptions. Consider first an example in which classical physics is used to determine the trajectory of a tennis ball, after a tennis player sends it back to the partner’s half of the court. The player’s consciousness is evidently involved—he or she sees the ball coming, chooses the moment and the angle, and finally strikes the ball with the racket. There are many aspects clearly outside the remit of classical mechanics, yet mechanics does not fail. Fortunately, to predict the ball’s trajectory one does not need to understand the mental processes that led to the force being exerted. Of the whole complex occurrence classical mechanics requires only the things firmly within the theory’s scope—the ball’s position and velocity, and the force acting at the time it is being hit.

Using this analogy, one can try to limit a complex act of Observer’s perception to its consequences in the inanimate *material* world. While the details of the act itself are outside the quantum theory’s scope, its consequences can be discussed and successfully used in practice. In order to do so, we will draw a distinction between Observers consciousness (fully outside our analysis) and his/her material memory (subject to our discussion) [20]. The act of registering an outcome will be seen as accompanied by a change in the state of the Observer’s memory’s, M, i.e., by production of a material record.

Furthermore, quantum theory will be expected to apply to all material objects regardless of their size and complexity. It will be able, therefore, to treat the change in the memory’s state without questioning how this change came about, just as classical mechanics does not need to question the chain of the player’s decisions, leading to the force being exerted on the ball. The appearance of this additional (memory’s) degree of freedom, entangled with the system, will enlarge the Hilbert space used in the calculation of Section 2.1, alter the degeneracies of the measured eigenvalues, and ultimately give different answers, depending on whether an Observer perceives his outcome or not. It will also follow that without producing such a record an act of observation “should not count”, just as a movement that misses the ball, or a movement contemplated yet not carried out, would have no effect on the ball’s trajectory.

We will need, therefore, to distinguish three different developments. First, an Observer *couples* his/her visible probe *D* to the studied quantum system *S* at t=τ (we consider all interactions to be instantaneous, and the system itself invisible to the naked eye). Then, at a $t_{r}>\tau$, he/she *registers* the state of the probe, which produces a record in his/her memory. Later still, at $t_{p}\geq t_{r}$ the Observer *perceives* the outcome, i.e., becomes aware of the impression left in his/her memory. This can happen at the time of the registration, or at a later time, when the Observer consults his/her memory again. The last step may seem redundant, but is necessary for our analysis. The probabilities in Section 2.1, we recall, refer to the moments the Observers are expected to *perceive* their outcomes.

### 2.4. An Example with only Two Participants

As an illustration, consider only two Observers, subsequently called *F* and *W*, a two-level system, *S*, two measuring devices (probes) $D^{F,W}$, visible to both Observers, and two sets of individual memories, $M_{F}$ and $M_{W}$. The experiment consists in preparing the composite system in a state $|\Phi_{0}\rangle \equiv |\mu_{0}^{W}\rangle |\mu_{0}^{F}\rangle |d_{0}^{W}\rangle \left|d_{0}^{F}\rangle \right|s_{0}\rangle$ at $t_{0}=0$. At $t=\tau^{F}>0$ *F* switches on a coupling which, after a brief interaction, entangles $D^{F}$ with *S*, using a particular basis $|s_{1,2}^{F}\rangle$ according to (for details see the Appendix A)

(12) $$\begin{array}{c} |d_{0}^{F}\rangle |s_{0}\rangle \to \langle s_{1}^{F}|{\hat{U}}^{S}\left(\tau^{F},t_{0}\right)|s_{0}\rangle |d_{1}^{F}\rangle |s_{1}^{F}\rangle +\langle s_{2}^{F}|{\hat{U}}^{S}\left(\tau^{F},t_{0}\right)|s_{0}\rangle \left|d_{2}^{F}\rangle \right|s_{2}^{F}\rangle , \end{array}$$

where ${\hat{U}}^{S}\left(\tau^{F},t_{0}\right)$ is the system’s evolution operator $\langle s_{j}^{F}|s_{i}^{F}\rangle =\delta_{ij}$, and $\langle d_{j}^{F}|d_{i}^{F}\rangle =\delta_{ij}$. Note that the device $D^{F}$ can be as complex as one wishes, bearing in mind that only two of its states $|d_{1}\rangle$ and $|d_{2}\rangle$, are involved in the experiment (For example, an outcome could be a sheet of paper, on which a printer may write “yes” or a “no”, but not T.S. Eliot’s Fourth Quartet. A similar situation occurs, for example, in cold matter physics, where only two states of a complex Rb atom are involved in the cooling experiment [21]).

Then at $t=t_{r}^{F}>\tau^{F}$, *F* “registers his result” (we might say “looks at the probe”), which, by means beyond our knowledge, changes the state of his memory according to

(13) $$\begin{array}{c} |\mu_{0}^{F}\rangle |d^{F}\rangle \to \langle d_{1}^{F}|d^{F}\rangle |\mu_{1}^{F}\rangle |d_{1}^{F}\rangle +\langle d_{2}^{F}|d^{F}\rangle \left|\mu_{2}^{F}\rangle \right|d_{2}^{F}\rangle , \end{array}$$

where $|d^{F}\rangle$ is any state of the F’s probe, and $\langle \mu_{j}^{F}|\mu_{i}^{F}\rangle =\delta_{ij}$. After that, at

(14) $$\begin{array}{c} t_{1}\equiv t_{p}^{F}\geq t_{r}^{F}, \end{array}$$

*F* accesses his memory (we do not need to know how), and becomes aware of (perceives) his outcome, a “yes${}^{F}$” or a “no${}^{F}$”.

Finally, the second Observer, *W*, is also able to measure the system, F’s probe, or their composite, by coupling his own visible device $D^{W}$ at $t=\tau^{W}>t_{r}^{W}$, registering the result at $t_{r}^{W}>\tau^{W}$, and becoming aware of his outcome (perceiving his outcome) $yes^{W}$ or a $no^{W}$ upon consulting his memory immediately after, at

(15) $$\begin{array}{c} t_{2}\equiv t_{p}^{W}=t_{r}^{W}+\epsilon ,\;\epsilon \to 0. \end{array}$$

For this *W* will need a probe with four orthogonal states $|d_{j}^{W}\rangle$, j=1,2,3,4, a four-state orthogonal basis for the composite *system + F’s probe*, for example,

(16) $$\begin{array}{c} |\phi_{1}^{W}\rangle =\alpha |s_{1}^{W}\rangle |d_{1}^{F}\rangle +\beta |s_{2}^{W}\rangle |d_{2}^{F}\rangle ,\;|\phi_{2}^{W}\rangle =\beta^{*}|s_{1}^{W}\rangle |d_{1}^{F}\rangle -\alpha^{*}\left|s_{2}^{W}\rangle \right|d_{2}^{F}\rangle ,\\ |\phi_{3}^{W}\rangle =\alpha |s_{1}^{W}\rangle |d_{2}^{F}\rangle +\beta |s_{2}^{W}\rangle |d_{1}^{F}\rangle ,\;|\phi_{4}^{W}\rangle =\beta^{*}|s_{1}^{W}\rangle |d_{2}^{F}\rangle -\alpha^{*}\left|s_{2}^{W}\rangle \right|d_{1}^{F}\rangle , \end{array}$$

where $\langle s_{j}^{W}|s_{i}^{W}\rangle =\delta_{ij}$ and ${\left|\alpha \right|}^{2}+{\left|\beta \right|}^{2}=1$, and a coupling that entangles W’s probe with the composite according to

(17) $$\begin{array}{c} |d_{0}^{W}\rangle |\phi \rangle \to \sum_{j=1}^{4}\langle \phi_{j}^{W}\left|\phi \rangle \right|d_{j}^{W}\rangle |\phi_{j}^{W}\rangle , \end{array}$$

where |ϕ〉 is any state of the composite (see Appendix A). Finally, *W* registers the state of his probe according to

(18) $$\begin{array}{c} |\mu_{0}^{F}\rangle |d^{W}\rangle \to \sum_{j=1}^{4}\langle d_{j}^{W}|d^{W}\rangle \left|\mu_{j}^{W}\rangle \right|d_{j}^{W}\rangle . \end{array}$$

To apply the rules of Section 2.1 we note that the acts of coupling, (12) and (17), and the acts of registering, (13) and (18), must be described by the evolution operators $\hat{U}$ in Equation (3), now acting in the Hilbert space of a composite *{spin + F’s probe + F’s memory + W’s probe + W’s memory}*. Different outcomes, perceived by *F* and *W*, are in one-to-one correspondence with distinct eigenvalues of operators ${\hat{Q}}^{F}$ and ${\hat{Q}}^{W}$, acting in the Hilbert spaces of F’s and W’s memories, respectively. Equation (5) will then give the probabilities of the possible outcomes, as perceived by the Observers. In the following we will assume that the probes and the memories, unlike the system, have none of their own dynamics, and consider several possible scenarios.

*Scenario (A): F does not register his outcome, and W registers and perceives his.* If so, F’s probe is coupled to the system as in Equation (12), but his memory remains unchanged, since (13) has not been applied. W’s perceived outcomes can be represented, for example, by an operator

(19) $$\begin{array}{c} {\hat{Q}}^{W}=|\mu_{1}^{W}\rangle \langle \mu_{1}^{W}\left|+2\right|\mu_{2}^{W}\rangle \langle \mu_{2}^{W}\left|+3[\right|\mu_{3}^{W}\rangle \langle \mu_{3}^{W}\left|+\right|\mu_{4}^{W}\rangle \langle \mu_{4}^{W}\left|\right], \end{array}$$

with the eigenvalues interpreted as $Q_{1}^{W}=1\to yes^{W}$, $Q_{2}^{W}=2\to no^{W}$, and $Q_{3}^{W}=3\to {\left\{not\;sure\right\}}^{W}$. By this we mean that the probabilities given by Equation (5) for the eigenvalues $Q_{i}^{W}$ are the actual odds on *W* saying that his experiment produced an outcome yes, no, or neither of the two. With *F*’s memory not involved, and remaining in its initial state $|\mu_{0}^{F}\rangle$, the basis states of the joint system *{system + F’s probe + W’s probe + W’s memory}* are conveniently chosen as

(20) $$\begin{array}{c} |q_{ijk}^{W}\rangle =|\mu_{k}^{W}\rangle \left|d_{j}^{W}\rangle \right|{\underline{\phi }}_{i}^{W}\rangle ,\;i,j,k=1,2,3,4, \end{array}$$

where $|{\underline{\phi }}_{i}^{W}\rangle \equiv {\hat{U}}^{S}\left(t_{p}^{W},\tau^{W}\right)|\phi_{i}^{W}\rangle$ and ${\hat{U}}^{S}\left(t_{p}^{W},\tau^{W}\right)$ is the system’s evolution operator. We will need the matrix elements, $\langle q_{ijk}^{W}|\hat{U}\left(t_{p}^{W},t_{0}\right)|\mu_{0}^{W}\rangle |d_{0}^{W}\rangle \left|d_{0}^{F}\rangle \right|s_{0}\rangle$, where $\hat{U}\left(t_{p}^{W},t_{0}\right)$ takes into account the developments (12), (17) and (18), but not (13). The matrix elements are easily evaluated, and we find

(21) $$\begin{array}{c} \langle q_{ij1}^{W}|\hat{U}\left(t_{p}^{W},t_{0}\right)|\mu_{0}^{W}\rangle |d_{0}^{W}\rangle \left|d_{0}^{F}\rangle \right|s_{0}\rangle =\delta_{i1}\delta_{j1}\left[\alpha^{*}A_{1}^{S}+\beta^{*}A_{4}^{S}\right]\\ \langle q_{ij2}^{W}|\hat{U}\left(t_{p}^{W},t_{0}\right)|\mu_{0}^{W}\rangle |d_{0}^{W}\rangle \left|d_{0}^{F}\rangle \right|s_{0}\rangle =\delta_{i2}\delta_{j2}\left[\beta A_{1}^{S}-\alpha A_{4}^{S}\right],\\ \langle q_{ij3}^{W}|\hat{U}\left(t_{p}^{W},t_{0}\right)|\mu_{0}^{W}\rangle |d_{0}^{W}\rangle \left|d_{0}^{F}\rangle \right|s_{0}\rangle =\delta_{i3}\delta_{j3}\left[\alpha^{*}A_{2}^{S}+\beta^{*}A_{3}^{S}\right],\\ \langle q_{ij4}^{W}|\hat{U}\left(t_{p}^{W},t_{0}\right)|\mu_{0}^{W}\rangle |d_{0}^{W}\rangle \left|d_{0}^{F}\rangle \right|s_{0}\rangle =\delta_{i4}\delta_{j4}\left[\beta A_{2}^{S}-\alpha A_{3}^{S}\right], \end{array}$$

where

(22) $$\begin{array}{c} A_{1}^{S}\equiv \langle s_{1}^{W}|{\hat{U}}^{S}\left(\tau^{W},\tau^{F}\right)|s_{1}^{F}\rangle \langle s_{1}^{F}\left|{\hat{U}}^{S}\left(\tau^{F},t_{0}\right)\right|s_{0}\rangle ,\\ A_{2}^{S}\equiv \langle s_{1}^{W}|{\hat{U}}^{S}\left(\tau^{W},\tau^{F}\right)|s_{2}^{F}\rangle \langle s_{2}^{F}\left|{\hat{U}}^{S}\left(\tau^{F},t_{0}\right)\right|s_{0}\rangle ,\\ A_{3}^{S}\equiv \langle s_{2}^{W}|{\hat{U}}^{S}\left(\tau^{W},\tau^{F}\right)|s_{1}^{F}\rangle \langle s_{1}^{F}\left|{\hat{U}}^{S}\left(\tau^{F},t_{0}\right)\right|s_{0}\rangle ,\\ A_{4}^{S}\equiv \langle s_{2}^{W}|{\hat{U}}^{S}\left(\tau^{W},\tau^{F}\right)|s_{2}^{F}\rangle \langle s_{2}^{F}\left|{\hat{U}}^{S}\left(\tau^{F},t_{0}\right)\right|s_{0}\rangle . \end{array}$$

**Figure 1:**
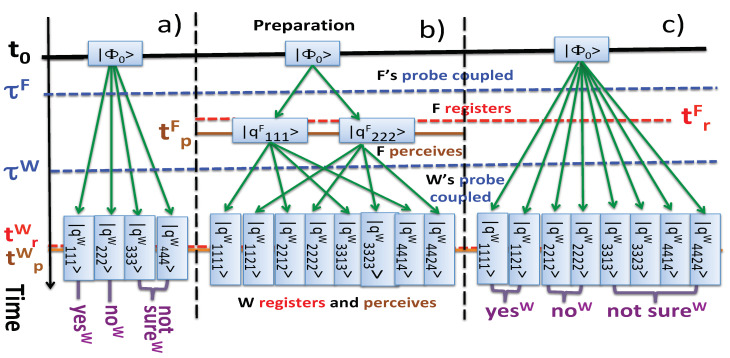
Virtual paths in the case (**a**) *F* does not register, nor perceive his outcome, and *W* perceives his outcome; (**b**) both *F* and *W* register and perceive their respective outcomes; (**c**) *F* only registers his outcome, and *W* registers and perceives his. In the scenario (**a**) *W* sees interference on his results. In (**b**,**c**) this interference is destroyed, since F’s memory carries a record of his outcome, even if it has not been perceived.

The four paths in the 64-dimensional Hilbert space of the composite *{system+F’s probe+W’s probe+W’s memory}* with non-zero amplitudes are shown in Figure 1a. Then, by (5), we have

(23) P1(yesW)=|α*A1S+β*A4S|2=|α|2|A1S|2+|β|2|A4S|2+2Re[α*βASA4s*],P1(noW)=|βA1S−αA4S|2,=|β|2|A1S|2+|α|2|A4S|2−2Re[α*βASA4s*]P1({notsure}W)=|α*A2S+β*A3S|2+|βA2S−αA43|2.

These three probabilities add up to one, as they should, since $\sum_{i=1}^{4}{\left|A_{i}^{S}\right|}^{2}=1$, ${\left|\alpha \right|}^{2}+{\left|\beta \right|}^{2}=1$, and $\langle s_{0}|s_{0}\rangle =1$.

*Scenario (B): Both F and W register and perceive their outcomes.* Next consider the case where both *F* and *W* become aware of their outcomes at the same time the outcomes become recorded in their memories,

(24) $$\begin{array}{c} t_{p}^{F}=t_{r}^{F}+\epsilon ,\;\epsilon \to 0. \end{array}$$

There are now two sets of possible outcomes and, according to Equations (2)–(5), we need the matrix elements of two operators, $\hat{U}\left(t_{p}^{F},t_{0}\right)$, which takes into account developments (12) and (13), and $\hat{U}\left(t_{p}^{W},t_{p}^{F}\right)$, which includes (17) and (18). To describe F’s relation with his memory, we will use an operator

(25) $$\begin{array}{c} {\hat{Q}}^{F}=|\mu_{1}^{F}\rangle \langle \mu_{1}^{F}\left|+2\right|\mu_{2}^{F}\rangle \langle \mu_{2}^{F}|, \end{array}$$

of which the eigenvalues are interpreted as $Q_{1}^{F}=1\to yes^{F}$, $Q_{2}^{F}=2\to no^{F}$. Since W’s probe and memory remain unchanged until $\tau^{W}>t_{p}^{F}$ we can choose eigenstates of ${\hat{Q}}^{F}$ to be

(26) $$\begin{array}{c} |q_{ijk}^{F}\rangle =|\mu_{0}^{W}\rangle |\mu_{k}^{F}\rangle |d_{j}^{F}\rangle \left|d_{0}^{W}\rangle \right|{\underline{s}}_{i}^{F}\rangle ,\;i,j,k=1,2, \end{array}$$

where $|{\underline{s}}_{i}^{F}\rangle \equiv {\hat{U}}^{S}\left(t_{p}^{F},\tau^{F}\right)|s_{i}^{F}\rangle$. To include F’s memory (no longer idle) in the calculation of W’s probabilities, we choose the eigenstates of ${\hat{Q}}^{W}$ in Equation (19) in the form

(27) $$\begin{array}{c} |q_{ijkl}^{W}\rangle =|\mu_{l}^{W}\rangle |\mu_{k}^{F}\rangle \left|d_{j}^{W}\rangle \right|{\underline{\phi }}_{i}^{W}\rangle ,\;i,j,l=1,2,3,4,\;k=1,2. \end{array}$$

With this, we have

(28) $$\begin{array}{c} \langle q_{ijk}^{F}|\hat{U}\left(t_{p}^{F},t_{0}\right)|\mu_{0}^{F}\rangle |d_{0}^{F}\rangle |s_{0}\rangle =\delta_{ij}\delta_{jk}\langle s_{i}^{F}\left|{\hat{U}}^{S}\left(\tau^{F},t_{0}\right)\right|s_{0}\rangle , \end{array}$$

(29) $$\begin{array}{c} \langle q_{i^{\prime }j^{\prime }k^{\prime }l^{\prime }}^{W}|\hat{U}\left(t_{p}^{W},t_{p}^{F}\right)|q_{ijk}^{F}\rangle =\delta_{i^{\prime }j^{\prime }}\delta_{j^{\prime }l^{\prime }}\delta_{k^{\prime }k}\langle \phi_{i^{\prime }}^{W}|{\hat{U}}^{S}\left(\tau^{W},\tau^{F}\right)\left|s_{i}^{F}\rangle \right|d_{i}^{F}\rangle , \end{array}$$

so that there are eight final states $|q_{i^{\prime }i^{\prime }k^{\prime }i^{\prime }}^{W}\rangle$, which can be reached from the initial $|\Phi_{0}\rangle$ via eight paths shown in Figure 1b. In particular we have

(30) $$\begin{array}{c} P\left(yes^{W},yes^{F}\right)=|\langle q_{1111}^{W}|\hat{U}\left(t_{p}^{W},t_{p}^{F}\right)|q_{111}^{F}\rangle \langle q_{111}^{F}|\hat{U}\left(t_{p}^{F},t_{0}\right)|\Phi_{0}{\rangle |}^{2}={\left|\alpha \right|}^{2}{\left|A_{1}^{S}\right|}^{2},\\ P\left(yes^{W},no^{F}\right)=|\langle q_{1121}^{W}|\hat{U}\left(t_{p}^{W},t_{p}^{F}\right)|q_{222}^{F}\rangle \langle q_{222}^{F}|\hat{U}\left(t_{p}^{F},t_{0}\right)|\Phi_{0}{\rangle |}^{2}={\left|\beta \right|}^{2}{\left|A_{4}^{S}\right|}^{2}, \end{array}$$

where the amplitudes $A_{1,2}^{S}$ are defined in Equation (22). Thus, the net probability of *W* perceiving a result $yes^{W}$,

(31) $$\begin{array}{c} P_{2}\left(yes^{W}\right)=P\left(yes^{W},yes^{F}\right)+P\left(yes^{W},no^{F}\right)={\left|\alpha \right|}^{2}|A_{1}^{S}{|}^{2}+{\left|\beta \right|}^{2}{\left|A_{4}^{S}\right|}^{2}, \end{array}$$

is different from the one in Equation (23), apparently changed by the fact that *F* had previously *perceived* his outcome.

*Scenario (C): F only registers his outcome, and W registers and perceives his.* We would have failed in our task of setting Observer’s consciousness aside, if a mere act of F’s perception could alter W’s statistics. There is, however, no danger of that happening, as seen from the example where F’s memory carries a record of his result, but *F* is not supposed to perceive it before the experiment is finished. According to the rules of Section 2.1, this case is formally different from the already discussed scenarios (A) and (B). Indeed, there is only one (*W*’s) set of perceived outcomes ($yes^{W}$, $no^{W}$, or ${\left\{not\;sure\right\}}^{W}$), and eight paths connecting $|\Phi_{0}\rangle$ with the final states $|q_{iiki}^{W}\rangle$, i=1,2,3,4, k=1,2, shown in Figure 1c. Now the probability of *W* perceiving an outcome $yes^{W}$ is

(32) $$\begin{array}{c} P_{3}\left(yes^{W}\right)=|\langle q_{1111}^{W}|\hat{U}\left(t_{p}^{W},t_{0}\right)|\Phi_{0}{\rangle |}^{2}+|\langle q_{1121}^{W}|\hat{U}\left(t_{p}^{W},t_{0}\right)|\Phi_{0}{\rangle |}^{2}={\left|\alpha \right|}^{2}|A_{1}^{S}{|}^{2}+{\left|\beta \right|}^{2}{\left|A_{4}^{S}\right|}^{2}, \end{array}$$

which is the same as $P_{2}\left(yes^{W}\right)$ in Equation (31), but differs from $P_{1}\left(yes^{W}\right)$ in Equation (23) by the absence of the interfering term 2Reαβ*A1SA4s*.

### 2.5. Feynman’S Photon, Future Possibilities, Destroyed Records, and Missed Opportunities

A brief summary is in order. A material record is carried by a system, to which the calculation of Section 2.1 ascribes a different orthogonal state, for each scenario, considered there. The world “material” is chosen to emphasise that the recording system is a material object, and nothing essential for calculating the probabilities is consigned to the Observer’s consciousness, where quantum theory has no jurisdiction. A simple example of such a record was given by Feynman in [17] where, in a double-slit experiment with electrons, a photon would end up in distinguishable (orthogonal) states, depending on the slit at which it was scattered by the passing particle. This alone will destroy the interference pattern on the screen even if the photon is never detected— *“At the end of the process you may say that you don’t want to look at the photon. That’s your business, but you still do not add the amplitudes...”* [17].

The case of the previous section are conceptually similar to the above example, with F’s memory playing the role of Feynman’s photon. Comparing the scenarios A and B, we note that quantum theory automatically accounts for the effect of previous (F’s) perceptions on the final (W’s) statistics *provided* the act of perception is accompanied by creation of a record in a material object representing Observers memory. As such, the memory is akin to any other object, carrying information about the observation’s outcome. For example, an Observer, not wishing to rely only on his/her memory, could decide to leave an additional note, for example, by preparing a spin up a given axis, if the result is a yes, or down that axis, if it is a no. In addition, he/she may decide to communicate the outcome to a friend, whose memory will be changed accordingly upon receiving this information. In all these cases, quantum mechanics will need only to take into account the records’ degrees of freedom, in order to be able to make the correct prediction using the rules of Section 2.1. Moreover, as our scenario C shows, the actual act of perception is not necessary. Even if a record, accessible to an Observer *in principle* [17] in future, is created by an inanimate device, the final statistics will look as if the outcome of and intermediate measurement had been experienced.

Feynman’s example may have interesting implications for an experiment where a macroscopic (F’s) probe becomes entangled with a small quantum system, such as an atom, or a spin, and *W* attempts to erase the information by entangling his probe to both F’s probe and the system, as in our scenario B. There, *W* failed to do so because of the persisting record in F’s memory. However, the same result would have been obtained even without *F* present, provided a single photon had been scattered by F’s probe, and then escaped W’s manipulations. Isolating a macroscopic device from all microscopic influences may be an extremely difficult task, even in the absence of a macroscopic environment, the presence of which is often assumed in decoherence theories [22].

One can still ask what would happen if *W* manages to entangle the system, F’s probe, F’s memory, the memory of F’s friend with whom F shared his outcome, and all the photons that were scattered during the experiment? At least, there is no formal contradiction. With all material records destroyed, the knowledge of what actually happened in the experiment will be irretrievably lost. This answer relies on an assumption we had to make, namely that no information about the physical world can be stored anywhere beyond the reach of the theory [20]. There is an agreement with purely classical reasoning—in a classical world, with *all* witnesses dead, and *all* records destroyed it should be impossible to know what did actually happen.

Finally, one may ask “did the information about F’s outcome exist before *W* destroyed all the records?” We recall that in the example of the previous section quantum mechanics provides probabilities for three hypothetical sequences of actual events. According to Feynman [17], a probability is desired for when the experiment is ’finished’, i.e., when *W* perceives his outcome at $t=t_{p}^{W}$. In the scenario (A), *F* had an opportunity to look at his probe and obtain his outcome at any $\tau^{F}<t<\tau^{W}$, but missed it. Were he to take this opportunity, we would be in the scenario (B). Finally, if a perceivable record were to be made without F’s knowledge, the theory would consider scenario (C), where there remains, at least *in principle* [17], a possibility of learning about the outcome in the future, at some $t>t_{p}^{W}$.

### 2.6. The Wigner’S Friend Problem

In a much cited paper [13], Wigner (W) considered the following situation. A quantum system *S*, initially is a state $\psi =\alpha |s_{1}\rangle +\beta |s_{2}\rangle$, is coupled to a probe *D*, and becomes entangled with it. The state of the composite *S+D* becomes $\phi =\alpha |d_{1}\rangle |s_{1}\rangle +\beta \left|d_{2}\rangle \right|s_{2}\rangle$, which is fully acceptable so far. If the probe is replaced by a conscious Observer, Wigner’s Friend (*F*), so that the states $|d_{1}\rangle$ and $|d_{2}\rangle$ correspond to him/her having seen the system in conditions $|s_{1}\rangle$ and $|s_{2}\rangle$, respectively, the situation changes. Should Wigner ask his Friend about the condition of *S* when he saw it, *F* has to give one of the two possible answers. The entangled state no longer makes sense, since it corresponds to neither $|s_{1}\rangle$, nor $|s_{2}\rangle$, and appears to imply that the *“friend was in a state of suspended animation”* [13] until asked by Wigner. The solution proposed in [13] was to suggest that the unsuitable state ϕ be replaced by a mixture ${\left|\alpha \right|}^{2}|d_{1}\rangle |s_{1}\rangle \langle d_{1}|\langle s_{1}{\left|+|\beta \right|}^{2}|d_{2}\rangle |s_{2}\rangle \langle d_{2}\left|\langle s_{2}\right|$, which now describes two exclusive alternatives available to F. This change, concludes Wigner, must be effected by Friend’s consciousness exerting influence upon the physical world, hence the necessity to make quantum equations of motion *“grossly non-linear if conscious beings enter the picture”* [13].

Let us reconsider the situation in a slightly different, yet equivalent form, after making one additional assumption. It is contrary to our experience that a person should be conscious of all information stored in his/her memory at all times. The sequence of events, about which *W* wants to make predictions, must therefore look like this. At $t=t_{0}$, *F* prepares the system and his probe, and keeps them apart until $t=t_{1}$. At $t_{1}$, he couples his probe to the system, looks at the probe, and consigns the outcome to his memory M. He then goes on thinking about unrelated matters, such as football or the state of the economy. After $t_{1}$, *S* and *D* may interact with each other, but not with M. At a $t=t_{2}>t_{1}$, *W* asks *F* about what he saw at $t=t_{1}$, so *F* has to consult his memory again, before coming up with an answer.

The situation is easily analysed by using the prescriptions of Section 2.1. We should consider a composite *{system + F’s probe + F’s memory}*. (If *F* were to make other records of his outcome, these would need to be included as well). As before, *F*’s perceived outcomes will be yes or no, *F*’s memory will couple to the probe as $|\mu_{0}\rangle |d\rangle \to \langle d_{1}\left|d\rangle \right|d_{1}\rangle |\mu_{1}\rangle +\langle d_{2}\left|d\rangle \right|d_{2}\rangle |\mu_{2}\rangle$, and the eigenvalues of *F*’s operator $\hat{Q}=|\mu_{1}\rangle \langle \mu_{1}\left|+2\right|\mu_{2}\rangle \langle \mu_{2}|$ will be interpreted as $Q_{1}=1\to yes$ and $Q_{2}=2\to no$. There are two perceived outcomes, one at $t=t_{1}$, when the result was first registered, and one at $t=t_{2}$, when the Friend needs to answer Wigner’s question. We require two sets of states for the composite,

(33) $$\begin{array}{c} |q_{ijk}^{\left(1\right)}\rangle =|\mu_{k}\rangle \left|d_{j}\rangle \right|s_{i}\rangle ,\;i,l,k,=1,2, \end{array}$$

and

(34) $$\begin{array}{c} |q_{ik}^{\left(2\right)}\rangle =|\mu_{k}\rangle |\varphi_{i}\rangle ,\;i,k=1,2,\;\varphi_{i}\equiv {\hat{U}}^{D+S}\left(t_{2},t_{1}\right)\left|d_{i}\rangle \right|s_{i}\rangle . \end{array}$$

In (33) we have assumed that F’s probe and the system do not interact before $t_{1}$, and the evolution operator ${\hat{U}}^{D+S}\left(t_{2},t_{1}\right)$ in (33) accounts for any interaction between *S* and *D* that may occur after *F* becomes aware of his outcome for the first time. For the amplitudes for the virtual paths $\left\{q_{i^{\prime }k^{\prime }}^{\left(2\right)}\leftarrow q_{ijk}^{\left(1\right)}\leftarrow \mu_{0}d_{0}s_{0}\right\}$ we have

(35) $$\begin{array}{c} A\left(q_{i^{\prime }k^{\prime }}^{\left(2\right)}\leftarrow q_{ijk}^{\left(1\right)}\leftarrow \mu_{0}d_{0}s_{0}\right)=\langle q_{i^{\prime }k^{\prime }}^{\left(2\right)}|\hat{U}\left(t_{2},t_{1}\right)|q_{ijk}^{\left(1\right)}\rangle \langle q_{ijk}^{\left(1\right)}|\hat{U}\left(t_{1},t_{0}\right)|\mu_{0}\rangle \left|d_{0}\rangle \right|s_{0}\rangle \\ \;\;=\delta_{kk^{\prime }}\delta_{ii^{\prime }}\delta_{jk}\delta_{ij}\langle s_{i}\left|{\hat{U}}^{S}\left(t_{1},t_{0}\right)\right|s_{0}\rangle \;\;\;\;\;\;\;\;\;\;\;\;\;\;\;\;\;\; \end{array}$$

There are two virtual paths with non-zero amplitudes, and the probabilities of *F* perceiving outcomes $yes^{1,2}/no^{1,2}$ at $t_{1,2}$ are

(36) $$\begin{array}{c} P\left(yes^{2},yes^{1}\right)=|A\left(q_{11}^{\left(2\right)}\leftarrow q_{111}^{\left(1\right)}\leftarrow \mu_{0}d_{0}s_{0}\right){|}^{2}=|\langle s_{1}|{\hat{U}}^{S}\left(t_{1},t_{0}\right){\left|s_{0}\rangle \right|}^{2},\\ P\left(yes^{2},no^{1}\right)={\left|A\left(q_{12}^{\left(2\right)}\leftarrow q_{222}^{\left(1\right)}\leftarrow \mu_{0}d_{0}s_{0}\right)\right|}^{2}=0,\;\;\;\;\;\;\;\;\\ P\left(no^{2},yes^{1}\right)={\left|A\left(q_{21}^{\left(2\right)}\leftarrow q_{111}^{\left(1\right)}\leftarrow \mu_{0}d_{0}s_{0}\right)\right|}^{2}=0,\;\;\;\;\;\;\;\;\\ P\left(no^{2},no^{1}\right)=|A\left(q_{22}^{\left(2\right)}\leftarrow q_{222}^{\left(1\right)}\leftarrow \mu_{0}d_{0}s_{0}\right){|}^{2}=|\langle s_{2}|{\hat{U}}^{S}\left(t_{1},t_{0}\right){\left|s_{0}\rangle \right|}^{2},\; \end{array}$$

which is the expected result. First, the Friend looks at his probe and consigns the outcome to his memory. When asked about it by Wigner, *F* consults the memory (or any other material record he may have produced) and gives an honest reply. Quantum mechanics duly takes notice of any records produced, and if the rules of Section 2.1 are accepted as its basic principle, no revision or extension of the existing formalism is required.

### 2.7. An Interference Gedankenexperiment

In a 1995 paper [18], Deutsch, very much in the spirit of Section 2.2, studied the “collapse” of the wave function, i.e., the process whereby a superposition $\sum c_{i}|\Phi_{i}\rangle$ goes into a single term, say, $|\Phi_{i_{0}}\rangle$, which corresponds to the actually observed value of the measured operator. In a slightly simplified form, the proposed experiment consists of coupling a system *S* to a probe *D*, then measuring an operator, able to detect whether the coupling took place, storing the outcome, and then reversing the system–probe evolution. In the scheme of [18], coupling of the probe is equivalent to “subsystem *D* measuring subsystem *S*”, and if this measurement is “complete”, the state of {S+D} would collapse [18], and will not be restored to its initial form by the reversed evolution. The experiment is finished with measuring S+D, in a different basis, so that the statistics of this last measurement would indicate whether the composite {S+D} ends up in a pure, or in a mixed state. In particular, one might ask whether knowing that the first measurement took place, but not its outcome, would have and effect on the statistics of the last measurement.

Unlike the author of [18], we are not interested in the virtues, or otherwise, of the Copenhagen and Everett’s many-world interpretations. Our aim is to demonstrate that by applying the rules of Section 2.1, we can get a definite answer without mentioning either of the two schools of thought directly. As before, we will employ two Observers, *F* and *W*, the former to establish that the measurement coupling did take place, and the latter to collect the final statistics. We will require three probes, *D*, $D^{F}$ and $D^{W}$, a two-level system *S*, and assume that only *S* has its own nontrivial dynamics. As before, at $t_{0}=0$ a composite *{system + probe + F’s probe + W’s probe + F’s memory + W’s memory}* is prepared in an initial state

(37) $$\begin{array}{c} |\Phi_{0}\rangle =|\mu_{0}^{W}\rangle |\mu_{0}^{F}\rangle |d_{0}^{W}\rangle |d_{0}^{F}\rangle \left|d_{0}\rangle \right|s_{0}\rangle . \end{array}$$

Then at $\tau >t_{0}$, *S* is entangled with the probe *D*, according to

(38) $$\begin{array}{c} |d_{0}\rangle |s\rangle \to \langle s_{1}\left|s\rangle \right|d_{1}\rangle |s_{1}\rangle +\langle s_{2}\left|s\rangle \right|d_{2}\rangle |s_{2}\rangle ,\;\;\;\;\;\\ \langle s_{j}\left|s_{i}\rangle =\delta_{ij},\;i=1,2,\;\langle d_{n}\right|d_{m}\rangle =\delta_{mn},\;m,n=0,1,2. \end{array}$$

Later still, at $t_{1}>\tau$, *F*’s probe is entangled with the composite {S+D}, and *F* immediately registers and perceives his outcome. This development is described as

(39) $$\begin{array}{c} |\mu_{0}^{F}\rangle |d_{0}^{F}\rangle |\phi \rangle \to |\mu_{1}^{F}\rangle |d_{1}^{F}\rangle \left[\alpha_{11}|d_{1}\rangle |s_{1}\rangle +\alpha_{22}\left|d_{2}\rangle \right|s_{2}\rangle \right]+\\ |\mu_{2}^{F}\rangle |d_{2}^{F}\rangle \left[\sum_{i=1}^{2}\sum_{j=0}^{2}\left(1-\delta_{j1}\delta_{i1}\right)\left(1-\delta_{j2}\delta_{i2}\right)\alpha_{ij}\left|d_{j}\rangle \right|s_{i}\rangle \right], \end{array}$$

where |ϕ〉 is an arbitrary state of the {S+D}, and $\alpha_{ij}\equiv \langle d_{j}\left|\langle s_{i}\right|\phi \rangle$, i=1,2, j=0,1,2. Equation (39) makes *F* a reliable witness—his memory is in a state $|\mu_{1}^{F}\rangle$ if the coupling (38) was applied (coupling on), and in a distinguishable state $|\mu_{2}^{F}\rangle$, if it was not (coupling off). After $t_{1}$, the evolution of *S* and *D*, up to the moment *F* couples his probe, is reversed. Until $t=t_{1}+\left(t_{1}-\tau \right)$ the system’s evolution operator is ${\hat{U}}^{S}\left(2t_{1}-\tau ,t_{1}\right)={\left[{\hat{U}}^{S}\right]}^{-1}\left(t_{1},\tau \right)$. At $t=2t_{1}-\tau$ the coupling (38) is reversed, to with

(40) $$\begin{array}{c} |d_{i}\rangle |s_{i}\rangle \to \left|d_{0}\rangle \right|s_{i}\rangle ,\;i=1,2. \end{array}$$

From $t=2t_{1}-\tau$ to $t_{2}=2t_{1}$, at which *W* perceives his outcome, we have ${\hat{U}}^{S}\left(t_{2},2t_{1}-\tau \right)={\left[{\hat{U}}^{S}\right]}^{-1}\left(\tau ,0\right)$. At $t_{2}=2t_{1}$, *W* may decide to explore the odds on finding the {S+D} in the initial state $|d_{0}\rangle |s_{0}\rangle$, $P(\mathrm{back}\;\mathrm{to}\;|d_{0}\rangle |s_{0}\rangle )$, by coupling and registering his probe according to

(41) $$\begin{array}{c} |\mu_{0}^{W}\rangle |d_{0}^{W}\rangle |\phi^{S+D}\rangle \to \langle s_{0}|\langle d_{0}\left|\right|\phi^{S+D}\rangle \times |\mu_{1}^{W}\rangle |d_{1}^{W}\rangle \left|s_{0}\rangle \right|d_{0}\rangle +\ldots , \end{array}$$

where $|\phi^{S+D}\rangle$ is an arbitrary state of the {S+D}, and we omitted the terms, containing the five remaining orthogonal states of the composite.

If the entanglement of probe *D* with *S* did not constitute a “complete measurement” (from (39) we know that the interaction between *S* and *D* did take place), the composite will be restored to $|d_{0}\rangle |s_{0}\rangle$ with a probability

(42) $$\begin{array}{c} P_{1}\left(\mathrm{back}\;\mathrm{to}\;\right|d_{0}\rangle |s_{0}\rangle )={\left[|\langle s_{1}|{\hat{U}}^{S}\left(\tau ,0\right)|s_{0}{\rangle |}^{2}+|\langle s_{2}|{\hat{U}}^{S}\left(\tau ,0\right){\left|s_{0}\rangle \right|}^{2}\right]}^{2}=\langle s_{0}|s_{0}\rangle^{2}=1. \end{array}$$

If a complete measurement takes place, at $t=t_{1}$, the “collapsed state” of the composite will have to be either ${\hat{U}}^{S}\left(t_{1},\tau \right)\left|s_{1}\rangle \right|d_{1}\rangle$, with a probability $|\langle s_{1}|{\hat{U}}^{S}\left(\tau \right){\left|s_{0}\rangle \right|}^{2}$, or ${\hat{U}}^{S}\left(t_{1},\tau \right)\left|s_{2}\rangle \right|d_{2}\rangle$, with a probability $|\langle s_{2}|{\hat{U}}^{S}\left(\tau \right){\left|s_{0}\rangle \right|}^{2}$, and W’s odds,

(43) $$\begin{array}{c} P_{2}\left(\mathrm{back}\;\mathrm{to}\;\right|d_{0}\rangle |s_{0}\rangle )=|\langle s_{1}|{\hat{U}}^{S}\left(\tau ,0\right)|s_{0}{\rangle |}^{4}+|\langle s_{2}|{\hat{U}}^{S}\left(\tau ,0\right){\left|s_{0}\rangle \right|}^{4}, \end{array}$$

will differ from $P_{1}$ in (42) by an interference term $P_{1}\left(\mathrm{back}\;\mathrm{to}\;\right|d_{0}\rangle |s_{0}\rangle )-P_{2}\left(\mathrm{back}\;\mathrm{to}\;\right|d_{0}\rangle |s_{0}\rangle )=2|\langle s_{1}|{\hat{U}}^{S}\left(\tau \right)|s_{0}{\rangle |}^{2}|\langle s_{1}|{\hat{U}}^{S}\left(\tau \right){\left|s_{0}\rangle \right|}^{2}$. Thus, the question is whether Equation (42) or Equation (43) will yield the correct answer, given that we *know* that the “subsystem *D* has measured the subsystem *S* at t=τ,” but do not know the measurement’s outcome.

**Figure 2:**
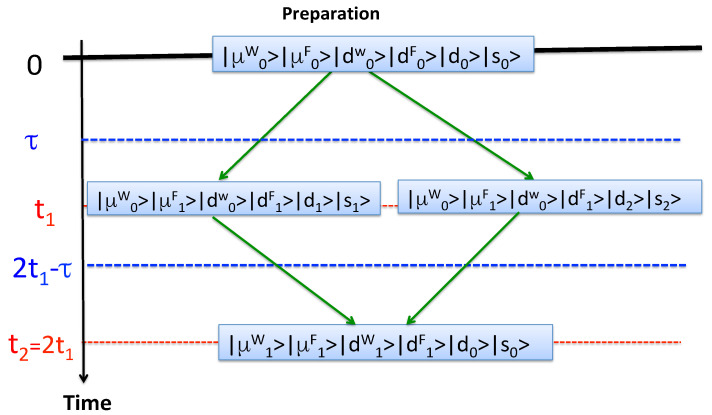
Two virtual paths in the interference experiment of Section 2.7. $|d_{j}^{F,W}\rangle$ and $|\mu_{k}^{F,W}\rangle$, j,k=0,1,2 are the states of F’s and W’s probes and memories, respectively.

The answer, easily found by applying the rules of Section 2.1, is shorter than the question it took us some time to formulate. Figure 2 shows two virtual paths connecting the initial and the final states with non-zero the amplitudes (i=1,2)

(44) $$\begin{array}{c} A_{i}\equiv \langle \mu_{1}^{W}|\langle \mu_{1}^{F}|\langle d_{1}^{W}|\langle d_{1}^{F}|\langle d_{0}|\langle s_{0}|\hat{U}\left(t_{2},t_{1}\right)|\mu_{0}^{W}\rangle |\mu_{1}^{F}\rangle |d_{0}^{W}\rangle |d_{1}^{F}\rangle \left|d_{i}\rangle \right|s_{i}\rangle \times \;\;\;\;\;\;\;\;\;\;\;\\ \langle \mu_{0}^{W}|\langle \mu_{1}^{F}|\langle d_{0}^{W}|\langle d_{1}^{F}|\langle d_{i}|\langle s_{i}|\hat{U}\left(t_{1},0\right)|\mu_{0}^{W}\rangle |\mu_{0}^{F}\rangle |d_{0}^{W}\rangle |d_{0}^{F}\rangle |d_{0}\rangle |s_{0}\rangle =\langle s_{0}|{\left[{\hat{U}}^{S}\left(\tau ,0\right)\right]}^{-1}|s_{i}\rangle \langle s_{i}\left|{\hat{U}}^{S}\left(\tau ,0\right)\right|s_{0}\rangle \end{array}$$

**Figure 3:**
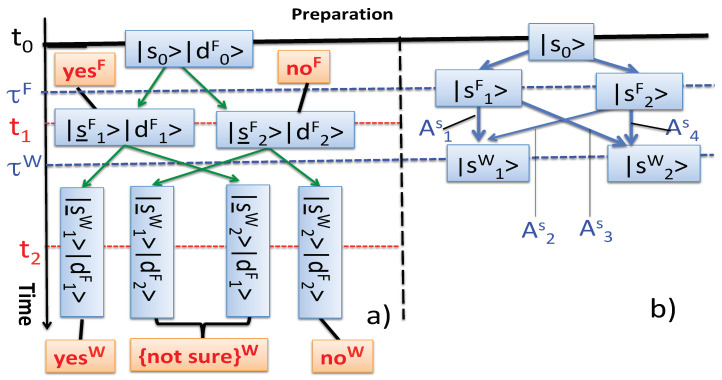
(**a**) Four real paths in the Hilbert space of *{system+F’s probe}*; (**b**) four virtual paths in the Hilbert space of the system only. Coupling to F’s probe does not change the values of the paths amplitudes $A_{i}^{S}$, but redirects the paths passing through $|s_{1}^{F}\rangle$ and $|s_{2}^{F}\rangle$ to different final states in the larger Hilbert space, thus turning them into exclusive alternatives.

F’s probe does not distinguish between the two scenarios shown in Figure 3, and neither can *F*, who may only know if the “measurement of S by D” did take place. Adding the amplitudes (44) we recover the correct result (42).

(45) $$\begin{array}{c} P(\mathrm{back}\;\mathrm{to}\;|d_{0}\rangle |s_{0}\rangle )=|A_{1}+A_{2}{|}^{2}=P_{1}\left(back\;to\;\right|d_{0}\rangle \left|s_{0}\rangle \right). \end{array}$$

This is the case of the Feynman’s photon, discussed in Section 2.5, with a difference that now the photon is always scattered into the same state, regardless of the slit chosen by the electron. Finding a scattered photon will signal the presence of a passing electron, but since no record of the path taken will be produced, an interfering pattern will be seen on the screen.

### 2.8. Reduction to the Hilbert Space of the Smallest System—The Von Neumann’S Boundary

In classical mechanics an observation is expected to yield information about the observed system “on its own”, i.e., not influenced by being observed. A vestige of this principle in the quantum case is evident, for example, from Equations (23), (36), or (44), where Observer’s probabilities are expressed in terms of the probability amplitudes $A_{i}^{S}$, referred to the system, uncoupled from the probes used to measure it. However, this is as far as the analogy goes. The amplitudes are combined differently, depending on whether the probes have been involved or not. This points towards a possibility of describing the measurements in a manner more economical than the one used up to now, namely by manipulating the system’s amplitudes $A_{i}^{S}$, without referring to the probes’ and the memory’ degrees of freedom, which occupy so much space, for example, in Figure 1.

The idea is by no means new. In [16] von Neumann pointed out that quantum theory can successfully avoid analysing an Observer, provided the boundary between the Observer and the observed system can be displaced arbitrarily.

For example, it can be placed between Observer and his/her memory, M, between the memory and the probe, *D*, or between the probe and the system *S*. The last option allows one to establish a direct correspondence between an Observer’s experience, and a particular property, which Observer’s theory ascribes to the system. Next we will apply this to a sequence of more than two measurements, L>2 relying, as before, on the rules of Section 2.1.

For an example, consider the case of Section 2.4 with a special choice α=1 and β=0, so that the four states in Equation (16) take the form

(46) $$\begin{array}{c} |\phi_{1}^{W}\rangle =|s_{1}^{W}\rangle |d_{1}^{F}\rangle ,\;|\phi_{2}^{W}\rangle =-|s_{2}^{W}\rangle |d_{2}^{F}\rangle ,\;|\phi_{3}^{W}\rangle =|s_{1}^{W}\rangle |d_{2}^{F}\rangle ,\;|\phi_{4}^{W}\rangle =-\left|s_{2}^{W}\rangle \right|d_{1}^{F}\rangle , \end{array}$$

and F’s probe, no longer affected by W’s measurement, continues to carry a record of the system’s condition as it was at $t=\tau^{F}$. The degrees of freedom describing F’s and W’s memories, and W’s probe, serve only to produce the Kronecker deltas in Equations (21) and (28), and are readily taken into account by considering the paths in the Hilbert space of a smaller composite *{system + F’s probe}*, shown in Figure 3a. The paths end in different orthogonal final states $|\phi_{j}^{W}\rangle$, j=1,2,3,4. According to the rules of Section 2.1 such paths cannot interfere, and can be endowed with probabilities, which are now the same for all three scenarios of Section 2.4 (cf. Equation (4)),

(47) $$\begin{array}{c} p\left(s_{1}^{W}d_{1}^{F}\leftarrow yes^{F}\leftarrow d_{0}^{F}s_{0}\right)=|A_{1}^{S}{|}^{2}=|\langle s_{1}^{W}|{\hat{U}}^{S}\left(\tau^{W},\tau^{F}\right)|s_{1}^{F}\rangle \langle s_{1}^{F}|{\hat{U}}^{S}\left(\tau^{F},t_{0}\right){\left|s_{0}\rangle \right|}^{2},\\ p\left(s_{1}^{W}d_{2}^{F}\leftarrow no^{F}\leftarrow d_{0}^{F}s_{0}\right)=|A_{2}^{S}{|}^{2}=|\langle s_{1}^{W}|{\hat{U}}^{S}\left(\tau^{W},\tau^{F}\right)|s_{2}^{F}\rangle \langle s_{2}^{F}|{\hat{U}}^{S}\left(\tau^{F},t_{0}\right){\left|s_{0}\rangle \right|}^{2},\\ p\left(s_{2}^{W}d_{1}^{F}\leftarrow yes^{F}\leftarrow d_{0}^{F}s_{0}\right)=|A_{3}^{S}{|}^{2}=|\langle s_{2}^{W}|{\hat{U}}^{S}\left(\tau^{W},\tau^{F}\right)|s_{1}^{F}\rangle \langle s_{1}^{F}|{\hat{U}}^{S}\left(\tau^{F},t_{0}\right){\left|s_{0}\rangle \right|}^{2},\\ p\left(s_{2}^{W}d_{2}^{F}\leftarrow no^{F}\leftarrow d_{0}^{F}s_{0}\right)=|A_{4}^{S}{|}^{2}=|\langle s_{2}^{W}|{\hat{U}}^{S}\left(\tau^{W},\tau^{F}\right)|s_{2}^{F}\rangle \langle s_{2}^{F}|{\hat{U}}^{S}\left(\tau^{F},t_{0}\right){\left|s_{0}\rangle \right|}^{2}. \end{array}$$

It is readily seen that the degrees of freedom describing F’s probe are also redundant since the r.h.s of Equation (47) contains only the references to the system that makes transitions between the states $|s_{0}\rangle$, $|s_{i}^{F}\rangle$, and $|s_{j}^{W}\rangle$. Thus, to calculate the probabilities (47) we may as well use a simpler diagram shown in Figure 3b. The diagram shows all virtual paths connecting $|s_{0}\rangle$ with $|s_{j}^{W}\rangle$ at $t=\tau^{W}$, and passing through $|s_{i}^{F}\rangle$ at $t=\tau^{F}$, when *F*’s coupling was applied. The only consequence of *F*’s probe being involved is that now the paths leading to the same final states $|s_{i}^{F}\rangle$ no longer interfere.

This amounts to a general rule. One can apply the prescription of Section 2.1 to the observed system, Observers’ probes, and their memories. However, the same probabilities can be obtained by applying the same prescription directly to the system, represented by virtual paths in its (smaller) Hilbert space. In this case, the “behind the scenes” presence of the Observers and their probes is translated into destruction of interference between otherwise indistinguishable system’s routes. This is, of course, the Feynman’s general rule for ascribing probabilities to distinguishable scenarios [17].

Observers’ memories, and their probes form the so-called von Neumann’s chains [16]. They are distinguished by a special type of interaction (A7), coupling them to all other degrees of freedom, which together constitute the observed “system” (cf. Section 2.4).

We note next that Equation (47) contains no mention of the times $t_{1}$ and $t_{2}$, at which *F* an *W* perceive their respective results, and refer instead to the times $\tau^{F}$ and $\tau^{W}$, at which F’s and W’s respective probes were coupled to the system. In the next Section we will discuss this lack of reference in more detail.

### 2.9. Unitary Evolution and the “in Principle” Principle

In the example of the previous Section, *F* may well decide not to register his probe before $t=t_{2}$, i.e., before the experiment is finished. This case is similar to the scenario *C* of Section 2.4 in that there is only one perceived result, (W’s) which needs to be taken into account when applying the rules of Section 2.1. In general, we can consider L−1 observers, all coupling their probes, $D^{\ell }$, to a system *S*, at $t=\tau_{\ell }$, ℓ=1,…L−1, according to (cf. Equation (A7))

(48) $$\begin{array}{c} |d_{0}^{\ell }\rangle |s\rangle \to \sum_{m_{\ell }=1}^{M_{\ell }}\left|d_{m_{\ell }}^{\ell }\rangle {\hat{\pi }}_{m_{\ell }}\right|s\rangle . \end{array}$$

but failing to register their conditions before $t=t_{L}$, when the last, *L*-th, Observer perceives his/her outcome. In principle, they could do it in the future, or maybe not do it at all.

Let the *L*-th observer measure a system’s quantity $S^{L}$, represented by an operator ${\hat{S}}^{L}=\left|s_{\nu }\rangle \langle s_{\nu }\right|$ whose two eigenvalues are interpreted as ${\tilde{S}}_{1}^{L}=1\to yes^{L}$, ${\tilde{S}}_{2}^{L}=0\to no^{L}$. The degrees of freedom, which describe the probe and the memory of the *L*-th Observer, can be left out of the calculation, as was discussed in the previous Section. For the probability of the *L*-th outcome “yes” we, therefore, have

(49) $$\begin{array}{c} P\left(yes^{L}\right)=\sum_{m_{1},\ldots m_{L-1}=0}^{M_{1},\ldots M_{L-1}}{\left|\langle s_{\nu }|\prod_{\ell =1}^{L-1}\langle d_{m_{\ell }}^{\ell }\left|\hat{U}\left(t_{L},t_{0}\right)\right|\Phi_{0}\rangle \right|}^{2},\;|\Phi_{0}\rangle \equiv |s_{0}\rangle \left|\prod_{\ell^{\prime }=1}^{L-1}\right|d_{0}^{\ell^{\prime }}\rangle , \end{array}$$

where the unitary evolution operator $\hat{U}\left(t_{L},t_{0}\right)$ accounts for the couplings (48), as well as for the free evolution of the system *S* between $\tau_{\ell }$ and $\tau_{\ell -1}$. The action of $\hat{U}\left(t_{L},t_{0}\right)$ is fairly simple. It decomposes a free system’s state ${\hat{U}}^{S}\left(t_{L},t_{0}\right)|s_{0}\rangle$ into $M_{1}\times M_{2}\ldots \times M_{L-1}$ in general non-orthogonal substates,

(50) $$\begin{array}{c} {\hat{U}}^{S}\left(t_{L},t_{0}\right)\left|s_{0}\rangle =\sum_{m_{1},\ldots m_{L-1}=1}^{M_{1},\ldots M_{L-1}}\right|s_{0}\left(m_{1},\ldots m_{L-1}\right)\rangle ,\;\;\;\;\\ |s_{0}\left(m_{1},\ldots m_{L-1}\right)\rangle \equiv {\hat{U}}^{S}\left(t_{L},\tau_{L-1}\right)\prod_{\ell =1}^{L-1}{\hat{\pi }}_{m_{\ell }}{\hat{U}}^{S}\left(\tau_{\ell },\tau_{\ell -1}\right)|s_{0}\rangle \end{array}$$

and then “tags” each substate by multiplying it by one of the orthogonal probes’ state $|d_{m_{1}}^{1}\rangle \ldots |d_{m_{L-1}}^{L-1}\rangle$, so that we have

(51) $$\begin{array}{c} |\Phi \left(t_{2}\right)\rangle \equiv \hat{U}\left(t_{L},t_{0}\right)\left|\Phi_{0}\rangle ={\hat{U}}^{S}\left(t_{L},t_{L-1}\right)\sum_{m_{1},\ldots m_{L-1}=1}^{M_{1},\ldots M_{L-1}}\left\{\prod_{\ell =1}^{L-1}|d_{m_{l}}^{\ell }\rangle \right\}\right|s_{0}\left(m_{1},\ldots m_{L-1}\right)\rangle . \end{array}$$

Now we can evaluate the matrix elements in Equation (49), or adopt the view of Section 2.2, and calculate $P(yes^{L})$ using the state $|\Phi (t_{2})\rangle$, obtained by a unitary evolution of $|\Phi_{0}\rangle$,

(52) $$\begin{array}{c} P\left(yes^{L}\right)={\mathrm{tr}}_{probes}\left[|s_{\nu }\rangle \langle s_{\nu }||\Phi \left(t_{2}\right)\rangle \langle \Phi \left(t_{2}\right)|\right]=\sum_{m_{1},\ldots m_{L-1}=1}^{M_{1},\ldots M_{L-1}}|\langle s_{\nu }{\left|s_{0}\left(m_{1},\ldots m_{L-1}\right)\rangle \right|}^{2}. \end{array}$$

Reversing the last argument of the previous Section, we note that if the remaining L−1. Observers, each measuring the system’s operators ${\hat{S}}^{\ell }=\sum_{m_{\ell }=1}^{M_{\ell }}{\tilde{S}}_{m_{\ell }}^{\ell }{\hat{\pi }}_{m_{\ell }}$, ℓ=1,…L−1, *did* register there probes and perceived the outcomes in the course of the experiment, i.e., at $\tau_{\ell }<t_{\ell }<t_{L}$, the probability would still be given by Equation (52),

(53) $$\begin{array}{c} P\left(yes^{L},{\tilde{S}}_{m_{L-1}}^{L-1}\ldots {\tilde{S}}_{m_{1}}^{1}\right)=|\langle s_{\nu }|s_{0}\left(m_{1},\ldots m_{L-1}\right){\rangle |}^{2}={\left|A^{S}\left(s_{\nu }\ldots .\leftarrow {\tilde{S}}_{m_{\ell }}^{\ell }\ldots \leftarrow s_{0}\right)\right|}^{2}. \end{array}$$

To arrive at Equation (53) we have, in fact, used the Feynman’s principle [17]: *“If you could”* in principle, *“distinguish the alternative final states (even though you don’t bother to do so), the total, final probability is obtained by calculating the” probability for each state (not the amplitude) and then adding them together.* We applied it to the system’s final states, $|s_{0}\left(m_{1},\ldots m_{L-1}\right)\rangle$, made distinguishable by the records, carried by the probes beyond the duration of the experiment, or, if one prefers, by the “tagging” evident in Equation (51). This saved us an effort of evaluating a large number of (mostly trivial) matrix elements connecting the states in the (large) Hilbert space of the composite *{system + all probes + all memories}*, as would be necessary if the rules of Section 2.1 were to be applied directly.

Thus, replacing the effect produced by the L−1 intermediate Observers by an uninterrupted unitary evolution (51) helps simplify the calculation. It may also please the reader, to whom the “collapse” of a wave function a nuisance, and a potential problem. His/her satisfaction would not, however, be complete. The need for the last, the *L*-th, Observer to become aware of his/her outcome implies projecting the overall state $|\Phi (t_{2})\rangle$ onto an orthogonal basis $\left(\prod_{\ell =1}^{L-1}|d_{m_{l}}^{\ell }\rangle \right)|s_{0}\left(m_{1},\ldots m_{L-1}\right)\rangle$, and there is nothing we can do about it. Quantum rules of Section 2.1 serve to predict statistical correlations between *at least two* Observers’ experiences (one of them disguised as “preparation”), and cannot be reduced further [19]. The content of these rules can, however, provide some insight into the matter. Calculation of matrix elements of operators between states in abstract Hilbert spaces (which is all that is required) does not rely on the concept of a constantly evolving “state”. Having a mental picture of such a state, and worrying about its fate after the *L*-th observer completes his/her observation, is just not necessary, if not futile, like wondering about what *actually* happens to the fictional personage of a novel, once the last page is turned.

### 2.10. Where We Agree and Disagree with the Consistent Histories Approach

Another method that uses the sequences of projectors similar to those in Equation (6) is the consistent histories approach (CHA) (see [14]), and the References therein), and we will briefly discuss it here. At first glance, the CHA could not be more different from our narrative. Indeed, while we aim at defining the probabilities of Observer’s perceptions, i.e., of “certain (subjective) observations” [16], the CHA, like [18], seeks a “framework for reasoning about the properties of closed physical system” [23], and gives no special role to a conscious Observer. According to the CHA, the probabilities $P(Q_{m_{L}}^{L}\ldots \leftarrow Q_{m_{\ell }}^{\ell }\ldots .\leftarrow Q_{m_{1}}^{1})$ can be ascribed to sequence projectors (12) (with L−1 changed to *L*), provided all partial evolutions of an initial state $|q_{0}\rangle$ result in mutually orthogonal states,

(54) $$\begin{array}{c} \langle q\left(Q_{m_{L}^{\prime }}^{L}\ldots \leftarrow Q_{m_{\ell }^{\prime }}^{\ell }\ldots .\leftarrow Q_{m_{1}^{\prime }}^{1}\right)|q\left(Q_{m_{L}}^{L}\ldots \leftarrow Q_{m_{\ell }}^{\ell }\ldots .\leftarrow Q_{m_{1}}^{1}\right)\rangle \\ =\delta_{m_{1}m_{1}^{\prime }}\ldots \delta_{m_{\ell }m_{\ell }^{\prime }}\ldots \delta_{m_{L}m_{L}^{\prime }}P_{CHA}\left(Q_{m_{L}}^{L-1}\ldots \leftarrow Q_{m_{\ell }}^{\ell }\ldots .\leftarrow Q_{m_{1}}^{1}\right), \end{array}$$

where (cf. Equation (6))

(55) $$\begin{array}{c} |q\left(Q_{m_{L}}^{L}\ldots \leftarrow Q_{m_{\ell }}^{\ell }\ldots .\leftarrow Q_{m_{1}}^{1}\right)\rangle \equiv \hat{U}\left(Q_{m_{L}}^{L}\ldots \leftarrow Q_{m_{\ell }}^{\ell }\ldots .\leftarrow Q_{m_{1}}^{1}\right)|q_{0}\rangle . \end{array}$$

Here we are not interested in the current discussion about the general merits of the CHA [24,25], and will comment only on the significance of Equations (54) and (55) for our own discourse.

The projectors ${\hat{\Pi }}_{m_{\ell }}^{\ell }$ in Equation (6) can stand for various things, and next we explore some of the possibilities.

(i) *The closed system, to which Equation (54) refers, contains L Observers, with their probes and memories.* If so, we have
  (56) $$\begin{array}{c} {\hat{\Pi }}_{m_{\ell }}^{\ell }=|\mu_{m_{\ell }}^{\ell }\rangle \langle \mu_{m_{\ell }}^{\ell }\left|⊗\right|d_{m_{\ell }}^{\ell }\rangle \langle d_{m_{\ell }}^{\ell }|⊗{\hat{\pi }}_{m_{\ell }}^{s}, \end{array}$$
  where the projector ${\hat{\pi }}_{m_{\ell }}^{s}$ refers to the system only, and the $\hat{U}\left(t_{\ell },t_{\ell -1}\right)$ in Equation (6) accounts for the system’s free evolution, the coupling of the *ℓ*-th probe according to
  (57) $$\begin{array}{c} |d_{0}^{\ell }\rangle |s\rangle \to \sum_{m_{\ell }=1}^{M_{\ell }}\left|d_{m_{\ell }}\rangle {\hat{\pi }}_{m_{\ell }}\right|s\rangle , \end{array}$$
  and for a similar coupling between the probe and the memory (cf. Equation (13)). In this case, the “consistency conditions” (54) are satisfied, and $P_{CHA}\left(Q_{m_{L}}^{L}\ldots \leftarrow Q_{m_{\ell }}^{\ell }\ldots .\leftarrow Q_{m_{1}}^{1}\right)$ is just the probability (5) for the *L* Observers to perceive their respective outcomes, at $t=t_{\ell }$.

(ii) *The closed system includes the L-th Observer, and L−1 probes, not registered before the experiment ends at $t=t_{L}$.* This implies ${\hat{\Pi }}_{m_{\ell }}^{\ell }=\left|d_{m_{\ell }}^{\ell }\rangle \langle d_{m_{\ell }}^{\ell }\right|⊗{\hat{\pi }}_{m_{\ell }}^{s}$, for ℓ=1,…L−1, and (56) for ℓ=L. For us the probabilities in Equation (54), identical to those in Equation (52), have no individual meaning, since the L−1 intermediate outcomes were not perceived. However, their sum yields the correct odds on the *L*-th observer perceiving an outcome $Q_{m_{L}}^{L}$,
  (58) $$\begin{array}{c} P\left(S_{m_{L}}^{L}\right)=\sum_{m_{1},\ldots m_{L-1}=1}^{M_{1},\ldots M_{L-1}}P_{CHA}\left(Q_{m_{L}}^{L}\ldots \leftarrow Q_{m_{\ell }}^{\ell }\ldots .\leftarrow Q_{m_{1}}^{1}\right). \end{array}$$
  We can also speculate about what would happen if the remaining L−1 Observers were to register their (protected) probes and perceive the outcomes after the experiment is finished, i.e., at $\tau_{\ell }>t_{L}$.

(iii) *The closed system includes only the system S and the consistency conditions (54) are satisfied.* Now we have ${\hat{\Pi }}_{m_{\ell }}^{\ell }={\hat{\pi }}_{m_{\ell }}^{s}$ and, since no Observers are present, have little to say about the probabilities $P_{CHA}\left({\tilde{S}}_{m_{L}}^{L}\ldots \leftarrow {\tilde{S}}_{m_{\ell }}^{\ell }\ldots .\leftarrow {\tilde{S}}_{m_{1}}^{1}\right)$. We can, however, speculate about what would happen if the Observers were to join in. With only the *L*-th Observer present, his/her odds on seeing an outcome corresponding to ${\tilde{S}}_{m_{L}}^{L}$ would be
  (59) $$\begin{array}{c} P\left(Q_{m_{L}}^{L}\right)=\sum_{m_{1},\ldots m_{L-1}=1}^{M_{1},\ldots M_{L-1}}P_{CHA}\left({\tilde{S}}_{m_{L}}^{L}\ldots \leftarrow {\tilde{S}}_{m_{\ell }}^{\ell }\ldots .\leftarrow {\tilde{S}}_{m_{1}}^{1}\right). \end{array}$$
  If the remaining L−1 Observers were to join in, ${\hat{\Pi }}_{m_{\ell }}^{\ell }\to |\mu_{m_{\ell }}^{\ell }\rangle \langle \mu_{m_{\ell }}^{\ell }\left|⊗\right|d_{m_{\ell }}^{\ell }\rangle \langle d_{m_{\ell }}^{\ell }|⊗{\hat{\pi }}_{m_{\ell }}^{s}$, the probabilities of their outcomes would be
  (60) $$\begin{array}{c} P\left(Q_{m_{L}}^{L}\ldots \leftarrow Q_{m_{\ell }}^{\ell }\ldots .\leftarrow Q_{m_{1}}^{1}\right)=P_{CHA}\left({\tilde{S}}_{m_{L}}^{L}\ldots \leftarrow {\tilde{S}}_{m_{\ell }}^{\ell }\ldots .\leftarrow {\tilde{S}}_{m_{1}}^{1}\right). \end{array}$$
  This is a “classical statistical” behaviour [26]—intermediate observations do not affect the final statistics, and the “which way?” question has an answer. As far as we are concerned, the consistency condition satisfied by the observed system, only indicates that no interference would be destroyed by observations of a particular type (but not by observations of *any* type), and some vestige of a classical behaviour can be retained.

(iv) *The closed system includes only the system S and the consistency conditions (54) are not satisfied.* This is, probably, where our disagreement with the CHA is most evident. In itself, the failure to satisfy the condition (54) means little to us, since we avoid to make statements about unobserved systems. We could, however, bring in all *L* Observers, which would return us to the case (i). In the enlarged Hilbert space of the composite, the consistency condition would be satisfied, since the non-orthogonal system’s substates $|q({\tilde{S}}_{m_{L}}^{L}\ldots \leftarrow {\tilde{S}}_{m_{\ell }}^{\ell }\ldots .\leftarrow {\tilde{S}}_{m_{1}}^{1})\rangle$ will acquire orthogonality upon multiplication by $|\mu_{m_{\ell }}^{\ell }\rangle ⊗|d_{m_{\ell }}^{\ell }\rangle$. Bringing in only the *L*-th observer will yield a different result, with his/her distribution $P_{\;\mathrm{one}}\left(Q_{m_{L}}^{L}\right)$ not being a marginal of $P_{\mathrm{all}}\left(Q_{m_{L}}^{L}\ldots \leftarrow Q_{m_{\ell }}^{\ell }\ldots .\leftarrow Q_{m_{1}}^{1}\right)$.

As a brief summary, our relations with the CHA approach can be described as follows.

Suppose the consistency conditions (54) are not satisfied for a closed system, not including Observers. For the CHA this is the end of the story—for us there is no story, since nothing is perceived. We can, however, add to the system Observers and their probes, all using the coupling (A8). Now we can calculate the probabilities, as in Section 2.1—but the CHA can calculate the same probabilities, now for a larger closed composite *{system+probes+memories}*.

Suppose next that the conditions (54) are satisfied, the CHA probabilities are available, and there are still no Observers present—this still means little to us. However, if the probes and Observers are added, the probabilities, calculated in Section 2.1 for the large composite, will be the same as the ones computed by the CHA, as an Equation (54) for the system only.

In other words, from our point of view, the CHA probabilities coincide with those predicted by the Feynman’s rules of Section 2.1, whenever the Observers are present, and are not particularly meaningful in the absence.

Finally, since it is up to the Observers to decide which measurements to make, there are many possibilities. For this reason, the CHA cannot single out a particular choice of the projectors ${\hat{\Pi }}_{m_{\ell }}^{\ell }$, without any prior knowledge of Observers’ intentions and must, therefore, favour all possible “frameworks” in equal measure. The CHA, sometimes deemed to be an *interpretation* of quantum theory, has often been criticised for the lack of guidance in choosing a particular “physical” representation, in which the calculation of the probabilities in (54) should be made in order to describe the actual experimental occurrences [24]. With the choices lying with the Observers, no a priori selection would, of course, be possible.

## 3. Conclusions

Like every empirical science, quantum mechanics relies on a set of axiomatic rules, which cannot be explained “from within the theory”. The rules need to be consistent, i.e., provide a plausible answer to any question within the theory’s area of expertise. A quantum discourse often contains issues, which prompt researchers to question its consistency. One such problem is the role and place of a conscious Observer (*O*). Views on the subject vary from assigning the consciousness an active role [13], to completely excluding the Observer from the narrative [14]. Neither of these extreme positions is particularly appealing. On the one hand, quantum mechanics is a theory by and for intelligent Observers. On the other hand, as a theory about the inanimate physical world [16], it is not obliged to provide an insight into the intelligence of its inventors.

In search of a compromise, the following analogy may be helpful. Tennis is undoubtably a game by and for conscious individuals. A ball would bounce off a wall, or off the player’s racket. The latter case is much more complex, since it involves the player, who sees the ball, takes a decision, and makes a deliberate action, all these developments beyond the reach of classical mechanics. Yet it does not prevent mechanics from calculating the ball’s trajectory, since the only input the theory needs for its predictions, is the force finally exerted on the ball.

Thus, one may want to look for something in an act of Observer’s perception, which would provide enough input for quantum theory to go on, without making it question the inner workings of Observer’s consciousness. Suppose an act of perception *always* results in a change in the state of a material object, destined to carry a record of the perceived outcome (and does not count if no such record is produced). Quantum theory can discuss material objects by assigning to them states in an abstract Hilbert space, and specifying their subsequent evolution. Observer’s memory may be such a material object, not to be confused from the Observer’s consciousness, which, we suspect, quantum theory cannot and should not analyse. Such an assumption does not contradict one’s everyday experiences. A person is not continuously aware of facts, and usually needs to consult the memory if asked. If the memory fails, he/she may need to consult a note, or a book—another material record, which can be looked at without altering its contents. The analogy is now obvious, although *O*’s memory, which is always at hand, might enjoy a particularly intimate relationship with *O*’s consciousness. Placed at the top of the von Neumann chain of devices, which connects *O*’s consciousness to the outside world, it is at the *“point at which we must say ‘And this is perceived by the observer’”* [16]. The details are of no concern to quantum theory, only interested in being able to treat the memory like any other physical object. To complete the analogy, consider a forgetful Observer, who, conscious of his impediment, decides to make a note for himself. Being a physicist, he takes a spin in a known initial state, and applies a magnetic filed to prepare it in a state up along the *z*-axis, if his experience was a yes, and down the axis, if it was a no. The spin is well protected from external influences, and its measurement along the *z*-axis at a later time, would remind *O* of what he has seen earlier. The fact that making such a note required certain conscious decisions on *O*’s part is of no consequence to quantum theory. If nothing else is changed, the only thing that matters to it is that the spin enters the picture, rotated by different angles in the different version of what happens.

By making the above assumption, we strike the required balance. On the one hand, quantum mechanics yields a probability of “an observer making a certain (subjective) observation” [16]. On the other hand, a theorist reasoning about what would be seen in an experiment, is able to treat all conscious participants as if they were degrees of freedom describing inanimate objects. Described in this way, the relationship between Observer’s consciousness and his/her memory bears a resemblance to the one between a computer’s operating system and its memory stored in its hard disc, but only in what relates to his/her experiences of the physical world. An Observer is free to devise experiments, write poetry, or pray to God—quantum mechanics cannot be a judge of these matters.

Evolution of an element of the von Neumann chain, connecting *O*’s memory and the measured system, need not be different from any other development, and yet not every interaction constitutes a measurement. A suitable coupling of a von Neumann’s type [16], which entangles Observer’s probe (*d*) and memory (μ) with the observed system (*s*) according to $|\mu_{0}\rangle |d_{0}\rangle |s\rangle \to \sum_{i}c_{i}|\mu_{i}\rangle \left|d_{i}\rangle \right|s_{i}\rangle$, is a particular case of a more general interaction, leading to $|\mu_{0}\rangle |d_{0}\rangle |s\rangle \to \sum_{i}\sum_{j}\sum_{k}c_{ijk}|\mu_{k}\rangle \left|d_{j}\rangle \right|s_{i}\rangle$. In the former special case, the boundary between the Observer and the observed system can be moved down the von Neumann chain [16] including the memory and the probe, and placed at the level of the system. It is then possible to say that intermediate observations “destroy interference between the system’s virtual paths”, in the spirit of Feynman’s analysis of the double slit experiment [17], to which we will return shortly.

A valid illustration of the above is the experiment of Section 2.4, where an Observer *F* measures a system using a probe, and later another Observer, *W*, measures a composite $\left\{system+F^{\prime }sprobe\right\}$. F may look at his probe, or look away, and what he did would make a difference to what *W* experiences. This somewhat surprising result can be explained without granting extra powers to F’s consciousness or intelligence, as was suggested, for example, in [13]. If the act of “looking” engages F’s memory, this additional degree of freedom must now be included into the calculation of W’s odds. At the level of the observed joint system, this amounts to the destruction of interference between the virtual paths in the Hilbert space of the composite, which, in turn, causes the disappearance of the interference term, otherwise present in W’s probabilities.

There are also broader consequences. Firstly, one would need to assume that the entire body of experimental knowledge about the physical world is contained in physical records, for example, in Observer’s memories and notes, and not in his/her consciousness, on the other side of the “observer/observed system divide” [16]. It follows logically that with all these material records destroyed, all knowledge of what actually happened in the physical world would be irretrievably lost. It follows also that in the example of Section 2.4, W’s measurement could in principle engage (difficult though it may be) also F’s memory, thus depriving *F* of the previously gained knowledge about what happened in his experiment. However, destruction of F’s record by W’s measurement does not lead to a formal contradiction. Wigner correctly objected to F’s consciousness being in “state of suspended animation” [13], since it contradicts our experience. This objection is lifted if the discussion is centred on F’s memory, which we expect to differ only in complexity from any other material object.

In his lectures [17], Feynman pointed out that a photon, scattered by the electron at one of the slits in a double slit experiment, should destroy the interference pattern even if it is never detected. Feynman also stressed that many, if not most situations in quantum mechanics are conceptually similar to the double slit example [17]. This is particularly true for our discussion. In the scenario *B* of Section 2.4 the role of Feynman’s photon was played by the Observer *F*, who, having looked at his probe, carries in his memory a record $yes^{F}/no^{F}$ of his outcome. This is sufficient for *W* to find no evidence of interference in his results, even without *F* telling him what his outcome was, or even with *F* having no recollection of the outcome. Not perfectly isolated from its environment, F’s memory may undergo a unitary evolution, so that his $yes^{F}/no^{F}$ records evolve into a pair of different orthogonal states, which now include the environment, and as such are no longer recognised by *F* as valid recollections. This does not change W’s situation, since his results depend on the presence oft two orthogonal states “tagging” the states of the system (cf. Equation (51)), and not on the nature of these states.

Feynman’s example [17] has other interesting consequences. Suppose that in the experiment of Section 2.4, half way up F’s von Neumann chain (the rest of the chain contains F’s retina, neurones, etc.) a printer prints either $yes^{F}$ or $no^{F}$ on a piece of paper. *W*, an extraordinarily able experimentalist, decides to entangle everything from the system to the printout, using a pair of states $[|yes^{F}\rangle \pm \left|no^{F}\rangle \right]/\sqrt{2}$, and a probe coupled to whole lab’s interior. F who is not in the room, which is sealed to the best of W’s ability, dedicates himself to evaluating W’s odds. He reckons that if the isolation of the lab is perfect, W’s result will contain an interference term. If, however, a single photon, missed by *W*, were to strike the printout on a dark spot and be absorbed, if the printed word is $yes^{F}$, or on a white spot and be reflected, if the word is $no^{F}$, W’s interference term will have to disappear. With several photons left inside, and given a large photon scattering cross section of the macroscopic equipment, *F* could think the second scenario to be more likely, and modify his calculation accordingly.

It is often assumed that quantum theory deals with things so small and delicate, that in any attempt to probe their condition, the condition is inevitably perturbed. In our previous example, this was not the case. A small and delicate photon appears to affect the state of something large, classical and fairly robust. This brings us to the second topic of our discussion. There is also a controversy surrounding the role and status of the quantum wave function, which stems from the desire to see the outcomes of an experiment in progress as a reflection on the real-time evolution of a certain physical state (or substance), associated with the observed system. This view was broadly outlined in Section 2.2. Moreover, anyone wishing to make unitary evolution the only basic principle of quantum theory immediately meets with the problem of “collapse” of a quantum state, be it of a pure, or of a mixed kind. A sudden decimation of the wave function after an Observer obtains a definite outcome cannot be described by the Schrödinger equation, and requires an additional “projection postulate” [16]. Accepting that a collapse is a physical phenomenon, prompts further questions about when and how exactly it occurs. One wishing to avoid these questions by preserving the integrity of the wave function against all odds, may decide to send its unused bits to parallel universes, as happens, for example, in the many-worlds interpretation of quantum mechanics [27]. A comparative analysis of these two viewpoints can be found, for example, in [18].

We argue that the above problems are artificial. They need not be solved, but can rather be dismissed once the appropriate terminology is adopted. It is possible to admit the rules of Section 2.1 as a basic principle, and consider Equations (6)–(9) of Section 2.2 to be their derivable consequences, serving mostly to simplify the calculations. Now Equations (2)–(5) are but a tool used by Alice, not taking part in the experiment herself, to reason about the odds on the outcomes perceived by the *L* intelligent Observers, were this experiment to be performed. Alice knows that the *ℓ*-th Observer’s inquiry about the system is represented by an operator ${\hat{Q}}^{\ell }$, and associates with the system a Hamiltonian ${\hat{H}}^{S}$. At the end of each run a sequence of Observer’s outcomes would be recorded, the numbers of identical records counted, and used to measure the probabilities that Alice is calculating in the comfort of her office. While doing so, Alice is not worried about the Observers’ consciousnesses, since in her calculation each participant is represented by the degrees of freedom of his/her material memory. It does not matter to Alice whether the Observers communicate with each other (provided their memories do not form a part of the measured system). Her job is to evaluate matrix elements of a unitary operator, $\hat{U}\left(t_{\ell }-t_{\ell -1}\right)=\exp\left[-i\hat{H}\left(t_{\ell }-t_{\ell -1}\right)\right]$, between the eigenstates of the ${\hat{Q}}^{\ell }$, representing the Observers’ measurements, multiply them, add the products as appropriate, and square the moduli of the resulting complex numbers. There is no mention of a wave function, expected to evolve in a continuous manner, nor any need to look for a home for its unwanted parts. However, Alice may have noticed that sometimes the formulae of Section 2.2 offer an easier way of calculating the probabilities, than the just described basic procedure. For example, instead of calculating the odds for *L* Observers she can evaluate, as we did in Section 2.9, the probabilities for a single Observer plus L−1 probes, already coupled, but as yet unregistered. This involves matrix elements of a single evolution operator (50) and is, for this reason, a simpler task. In the process, Alice may begin to put more faith in the universal value of unitary evolution, but does not have to do so. The rules of Section 2.1 serve only to a establish statistical correlation between at least two Observers’ experiences, and cannot be reduced further [19]. In the above example, the *L*-th Observer’s definite outcome would “ collapse” the state, making Alice wonder about the destiny of the rest of the so far unitary evolved wave function. However, as we said, this is by no means necessary. Alice could as well proclaim “the experiment finished, the desired probability evaluated” (cf. [17]), and close her notebook. She would refuse to answer Bob’s question “what happened to the system after that?” However, if asked instead “what would be the results of an L+1-st Observer, who decides to join the experiment at some $t_{L+1}>t_{L}$?”, she would reopen the notebook, make a new calculation for the *entire* new series of outcomes, $\left\{Q_{m_{L+1}}^{L+1}\ldots \leftarrow Q_{m_{\ell }}^{\ell }\ldots .\leftarrow Q_{m_{0}}^{0}\right\}$, and then close it again.

In summary, we found elementary quantum mechanics consistent, in the sense of being able to provide an unambiguous answer at least in the hypothetical situations considered in this work. The “minimalist” view [19], advocated here, comes at a price of making certain additional assumptions. In particular, the theory is deemed to predict statistical correlations between at least two of the Observer’s *“subjective observations”* [16], accompanied by producing, or consisting in consulting’s a record in the Observer’s material memory. With the line between Observer’s consciousness and the physical world drawn at the memory’s level, Feynman’s *general principles* [17] need to be applied to the *entire* duration of the experiment. The focus is thereby shifted from a continuously evolving wave function to the transition amplitudes (2)–(3), seen as mere tools of human reasoning. This helps one avoid unfruitful (in our opinion) discussions about the exact moment in which a quantum state “collapses” [18], or whether the unused parts of the state found their use in parallel worlds [27].

Expected restrictions on potential applications of quantum theory are also considerable. Quantum mechanics is not expected to make statements about human consciousness, and cannot explain how consciousness addresses the memory, or retrieves the memorised information from it. With the probabilities referring to humans experiences (actual, or possible *in principle*), further questions arise about the theory’s retroductive powers (see e.g., [25]), as well as about such global concepts as the wave function of the entire Universe.

Finally, we note certain parallels with the relational quantum mechanics of Rovelli [28]. We will return to these issues in our future work, and conclude with a picture, in which various Alices and Bobs perform experiments of their choice, perceive the outcomes, memorise and forget, produce and destroy records of their outcomes, wittingly or unwittingly, and share or not their experiences with each other. In the meantime, Carols (the roles can of course be exchanged) are evaluating the likelihoods of Alices’ and Bobs’ outcomes, taking into account only the changes their actions may produce in the inanimate physical world. 

## Abbreviations

The following abbreviations are used in this manuscript:

UP	Uncertainty Principle
CHA	Consistent Histories Approach

## Appendix A. Coupling a Probe to the System

Consider a system *S* in a Hilbert space of a dimension *N*, and an operator with M≤N distinct eigenvalues ${\tilde{S}}_{m}$, (Δ(x−y)=1, if x=y, and 0 otherwise)

(A1) $$\begin{array}{c} \hat{S}=\sum_{n=1}^{N}|s_{n}\rangle S_{n}\langle s_{n}|=\sum_{m=1}^{M_{L}}{\tilde{S}}_{m}\sum_{n=1}^{N}\Delta ({\tilde{S}}_{m}-\langle s_{n}|\hat{S}|s_{n}\rangle )|s_{n}\rangle \langle s_{n}|\equiv \sum_{m=1}^{M_{L}}{\tilde{S}}_{m}{\hat{\pi }}_{m}. \end{array}$$

Consider also a probe (a von Neumann’s pointer), a massive particle in one dimension, with a coordinate *q*, and a momentum operator (ℏ=1) $\hat{p}$$\langle q^{\prime }\left|\hat{p}\right|q\rangle =-i\delta \left(q-q^{\prime }\right)\partial_{q}$. The Hamiltonian, coupling the system to the pointer will be ${\hat{H}}_{int}=\hat{p}\hat{S}\delta \left(t-\tau \right)$, so that the evolution operator over a period τ−ϵ<t<τ+ϵ is $\hat{U}\left(\tau +\epsilon ,\tau -\epsilon \right)=\exp\left(-i\hat{p}\hat{S}\right)$ is given by

(A2) $$\begin{array}{c} \hat{U}\left(\tau +\epsilon ,\tau -\epsilon \right)=\sum_{n=1}^{N}\int dq|q+S_{n}\rangle |s_{n}\rangle \langle s_{n}\left|\langle q\right|. \end{array}$$

Initially, the system and the pointer are described by a product state $|\Phi_{0}\rangle =\left|s\rangle \right|G\rangle$, where

(A3) $$\begin{array}{c} |s\rangle =\sum_{n=1}^{N}c_{n}|s_{n}\rangle ,\;\mathrm{and}\;\left|G\rangle =\int dqG\left(q\right)\right|q\rangle , \end{array}$$

so that

(A4) $$\begin{array}{c} \hat{U}\left(\tau +\epsilon ,\tau -\epsilon \right)|\Phi_{0}\rangle =\sum_{n=1}^{N}c_{n}\left|s_{n}\rangle \right|G\left(S_{n}\right)\rangle =\;\;\;\;\;\;\;\;\\ \sum_{m=1}^{M}|G\left({\tilde{S}}_{m}\right)\rangle \sum_{n=1}^{N}\Delta ({\tilde{S}}_{m}-\langle s_{n}|\hat{S}|s_{n}\rangle )c_{n}|s_{n}\rangle =\sum_{m=1}^{M}\left|G\left({\tilde{S}}_{m}\right)\rangle {\hat{\pi }}_{m}\right|s\rangle . \end{array}$$

where |G(Z)〉≡∫dqG(q−Z)|q〉. Let G(q) be a Gaussian of a width Δq,

(A5) $$\begin{array}{c} G\left(q\right)=C\exp\left(-q^{2}/\Delta q^{2}\right),\;\int {\left|G\left(q\right)\right|}^{2}dq=1, \end{array}$$

and send Δq to zero, so that $\langle G\left({\tilde{S}}_{m^{\prime }}\right)|G\left({\tilde{S}}_{m}\right)\rangle \to \delta_{mm^{\prime }}$. Although the probe’s Hilbert space has of infinite dimensions, we will only need its M+1 orthogonal states,

(A6) $$\begin{array}{c} |d_{0}\rangle \equiv \left|G\rangle ,\;\mathrm{and}\;\right|d_{j}\rangle \equiv |G\left({\tilde{S}}_{j}\right)\rangle ,\;j=1,\ldots M. \end{array}$$

and will describe the application of the coupling (A2) by saying that *“ the system is coupled to (entangled with) the probe according to”*

(A7) $$\begin{array}{c} |d_{0}\rangle |s\rangle \to \sum_{m=1}^{M}\left|d_{m}\rangle {\hat{\pi }}_{m}\right|s\rangle . \end{array}$$

The coupling (A2) can be reversed by applying $-{\hat{H}}_{int}$, the action of which is defined as

(A8) $$\begin{array}{c} |d_{j}\rangle {\hat{\pi }}_{j}\left|s\rangle \to \right|d_{0}\rangle {\hat{\pi }}_{j}|s\rangle . \end{array}$$
